# Encapsulation of Platinum Prodrugs into PC7A Polymeric Nanoparticles Combined with Immune Checkpoint Inhibitors for Therapeutically Enhanced Multimodal Chemotherapy and Immunotherapy by Activation of the STING Pathway

**DOI:** 10.1002/advs.202205241

**Published:** 2022-12-11

**Authors:** Xiangjie Gao, Guanxiong Lei, Bin Wang, Zhong Deng, Johannes Karges, Haihua Xiao, Donghui Tan

**Affiliations:** ^1^ Affiliated Hospital of Xiangnan University Chenzhou Hunan Province 423000 China; ^2^ Xiangnan University Chenzhou Hunan Province 423000 China; ^3^ Key Laboratory of Medical Imaging and Artificial Intelligence of Hunan Province Chenzhou Hunan Province 423000 China; ^4^ Hunan Engineering Research Center of Advanced Embedded Computing and Intelligent Medical Systems, Xiangnan University Chenzhou Hunan Province 423000 China; ^5^ Beijing National Laboratory for Molecular Sciences State Key Laboratory of Polymer Physics and Chemistry Institute of Chemistry Chinese Academy of Sciences Beijing 100190 China; ^6^ Faculty of Chemistry and Biochemistry Ruhr‐University Bochum Universitätsstrasse 150 44780 Bochum Germany

**Keywords:** immunotherapy, nanoparticle, oxaliplatin, polymer with a cyclic seven‐membered ring (PC7A), stimulator of interferon genes (STING)

## Abstract

Tumor immunotherapy has emerged as one of the most promising therapeutic methods to treat cancer. Despite its clinical application, the immunosuppressive tumor microenvironment compromises the therapeutic efficiency of this technique. To overcome this limitation, many research efforts have been devoted to the development of agents that reprogram the immunosuppressive tumor microenvironment through novel mechanisms. Over the last decade, compounds that intervene through the immunogenic stimulator of interferon genes (STING) pathway have emerged with potential for clinical development. Herein, the encapsulation of chemotherapeutic platinum complexes with a polymer with a cyclic seven‐membered ring (PC7A)‐based polymer into pH‐responsive nanoparticles for multimodal therapeutically enhanced chemotherapy and immunotherapy is presented. This study represents the first nanomaterial with a dual activation mechanism of the STING pathway through DNA fragmentation as well as PC7A binding. The combination of these nanoparticles with immune checkpoint inhibitors demonstrates to nearly fully eradicate a colorectal tumor inside the mouse model by chemotherapy and immunotherapy using the STING pathway.

## Graphical abstract

Despite recent advancements in the field, the immunosuppressive tumor microenvironment is considered as the primary factor for the reduced therapeutic effect of tumor immunotherapy. To overcome this limitation, herein, the encapsulation of a chemotherapeutic platinum complex with a PC7A‐based polymer into pH‐responsive nanoparticles for multimodal therapeutically enhanced chemotherapy and immunotherapy upon dual activation of the STING pathway is presented.

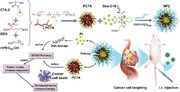

## 1 Introduction

Cancer has emerged as one of the deadliest diseases worldwide. Traditional treatment modalities involve a combination of techniques whereby the primary tumor is removed in a surgical procedure and the patient is further treated by immunotherapy, radiotherapy, or chemotherapy. Despite the success achieved with these approaches, many treatments are associated with severe side effects and more worryingly, an increasing number of drug‐resistant tumors, relapses, and metastatic tumors are reported. Among the most promising therapeutic techniques to treat challenging tumors, much efforts have been devoted to the improvement of immunotherapeutic strategies.^[^ ^1^ ^]^ To date, the immunosuppressive microenvironment is the leading cause of the poor therapeutic efficiency of immunostimulating agents.^[^ ^2^ ^]^ To overcome this limitation, many research efforts have been focused on agents which are able to reprogram the immunosuppressive microenvironment.^[^ ^3^ ^]^ As one of the most promising strategies to enhance the immunotherapeutic effect, recent studies have indicated the activation of the stimulator of interferon genes (STING) pathway.^[^ ^4^ ^]^

Studies have shown two different distinguished mechanisms to active the STING pathway. The first mechanism involves the use of DNA damaging agents (i.e., radiation therapy, chemotherapy).^[^ ^5^ ^]^ The generated DNA fragments are recognized and bound by cyclic guanosine phospho‐adenylate (c‐GAS) in the cytoplasm, that further catalyzes the formation of cyclic guanosine monophosphate–adenosine monophosphate (c‐GAMP).^[^ ^6^ ^]^ Followingly, c‐GAMP is able to bind to STING proteins and induce their dimerization,^[^ ^7^ ^]^ resulting in the activation of the STING pathway through the production of type‐I interferons and proinflammatory factors which can stimulate the maturation of dendritic cells.^[^ ^8^ ^]^ This mechanism allows for an activation of the immune response of the organism as well as the inhibition of the immunosuppressive characteristics of the tumor microenvironment. Based on the involvement of cyclic guanosine phospho‐adenylate, this mechanism is also referred to as the cGAS‐STING pathway.^[^ ^9^ ^]^ Despite preliminary investigations, the use of currently studied compounds for clinical application remains limited due to 1) poor accessibility and bioavailability of c‐GAMP, 2) poor DNA fragmentation of the therapeutic agent, 3) poor water solubility and stability of the therapeutic agent, and 4) low cancer/tumor targeting properties of the therapeutic agent.^[^ ^10^ ^]^ Capitalizing on this, there is a need for novel therapeutic compounds/materials with an enhanced therapeutic effect and improved pharmacological properties.

The second mechanism for activation of the STING pathway involves the use of the polymer‐based material PC7A. The seven‐membered tertiary amine moiety could directly bind to the STING protein and therefore activate the immune response.^[^ ^11^ ^]^ While preliminary studies have demonstrated the successful immune activation with PC7A, the use for clinical application of this polymer‐based material is limited due to 1) the slow immune activation (in particular inside animal models in comparison to c‐GAMP of the cGAS‐STING pathway), and 2) the slow biodegradability of the therapeutic PC7A polymer.^[^ ^12^ ^]^ To enhance the therapeutic efficiency, there is a need for the combination of the PC7A‐based immune activation with other therapeutic agents and mechanisms.

**Figure 1:**
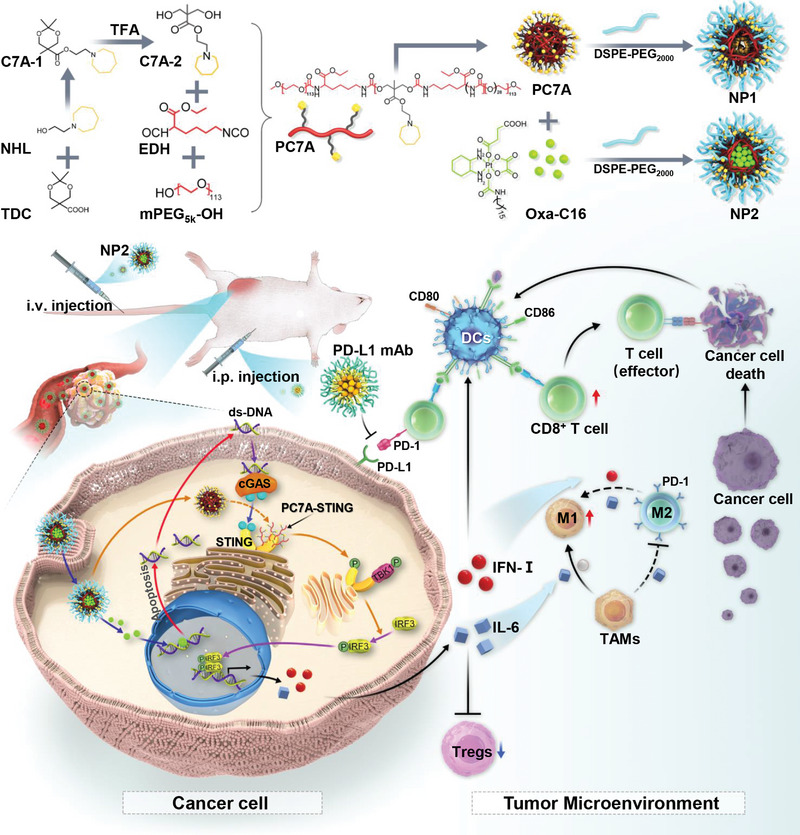
Schematic illustration showing the preparation and biological mechanism of action of the encapsulation of platinum complexes with the PC7A polymer into multimodal nanoparticles for chemotherapy and immunotherapy using the STING pathway.

To overcome these limitations, herein, the encapsulation of chemotherapeutic platinum complexes with a PC7A‐based polymer into pH‐responsive, biodegradable nanoparticles (**NP2**) for multimodal therapeutically enhanced chemotherapy and immunotherapy by dual activation of the STING pathway is reported. The proposed nanomaterial was designed to dual activate the STING pathway by DNA fragmentation/cGAS activation as well as PC7A binding. While the nanoparticles remained stable under physiological conditions, the nanomaterial quickly degraded in the acidic tumor microenvironment. Insights into the mechanism of action revealed that the nanoparticles were able to intervene combined by generation of DNA damage through chemotherapy as well as by the systemic induction of an immune response through immunotherapy using the STING pathway (**Figure** **1**). The STING pathway could promote the maturation of dendritic cells and improve the cross‐presentation of dendritic cells, ultimately generating memory T cells for long‐lasting enhanced antitumor immunity. The combination of the therapeutic properties of the nanoparticles with immune checkpoint inhibitors demonstrated to almost fully eradicate a colorectal tumor inside the mouse model by chemotherapy and immunotherapy using the STING pathway.

## 2 Results and Discussion

### 2.1 Preparation and Characterization

Previous studies have indicated that the PC7A‐based polymers could activate the STING pathway and therefore promote the immune response in the organism. To further enhance the therapeutic anticancer effect, herein, a multimodal nanoparticle formulation for enhanced combined chemotherapy and immunotherapy was designed. The strategy is based on the 1) incorporation of PC7A which acts as potent STING agonists into the polymer backbone to promote the immune response; 2) encapsulation of a therapeutic platinum(IV) complex which is able to cross‐link DNA and cause significant DNA damage; 3) pH sensitivity to the tumor microenvironment to release the therapeutic payload; 4) amphiphilic nature of the polymer backbone to enable self‐assembly into nanoparticles; 5) terminal polyethylene glycol/phospholipid functionalization to enhance the physiological stability and water solubility; and 6) selective tumor accumulation due to the enhanced permeability and retention effect of the nanomaterial. Notably, the structure of the here designed polymeric material is significantly different from previously reported PC7A‐based polymers. Herein, ethyl‐2,6‐diisocyanatohexanoate was incorporated as an aliphatic linker in the polymer backbone and the terminal ends of the polymer were capped with polyethylene glycol groups, allowing for a stronger amphiphilic character and therefore enhanced properties for self‐assembly into nanoparticles. Additionally, in this study, the C7A monomeric unit was included to a lesser extent. These differences are expected to significantly change the physicochemical properties as well as influence the immune‐activation of the material. The combination of PC7A as a STING agonist and the platinum complex oxaliplatin as a DNA damaging therapeutic compound will allow for an enhanced combined chemo‐ and immunotherapeutic effect upon dual activation of the STING pathway.

The C7A‐1 precursor was synthesized by ester coupling of 2,2,5‐trimethyl‐1,3‐dioxane‐5‐carboxylic acid and 2‐(azepan‐1‐yl)ethanol using 1‐ethyl‐3‐(3‐dimethylaminopropyl)carbodiimide. The 1,3‐dioxane heterocycle of C7A‐1 was opened to form C7A‐2 upon treatment with trifluoroacetic acid. The hydroxy groups of the monomer C7A‐2 and the isocyanate groups of ethyl‐2,6‐diisocyanatohexanoate were conjugated to form a polymer that was terminally functionalized with polyethylene glycol to generate PC7A (Figure 1). The precursors C7A‐1 and C7A‐2 (Figure S1, Supporting Information) and the polymer PC7A (Figure S2, Supporting Information) were characterized by ^1^H and ^13^C‐NMR spectroscopy. Using gel permeation chromatography, the polymer was found with an average weight of 23 500 Da and a uniform distribution with a polydispersity index of 1.22 (Figure S2, Supporting Information). This indicates that on average 27.8 monomeric units of C7A were incorporated in each polymer chain. The platinum(II) chemotherapeutic agent oxaliplatin (**Oxa**) was axially functionalized with a hydrophobic long fatty acid chain and a succinic acid to form the platinum(IV) prodrug **Oxa‐C16** according to a previously reported protocol by oxidation of **Oxa** with hydrogen peroxide and subsequent functionalization of the axial positions.^[^ ^13^ ^]^ The metal complex was characterized by ^1^H spectroscopy (Figure S3, Supporting Information).

**Figure 2:**
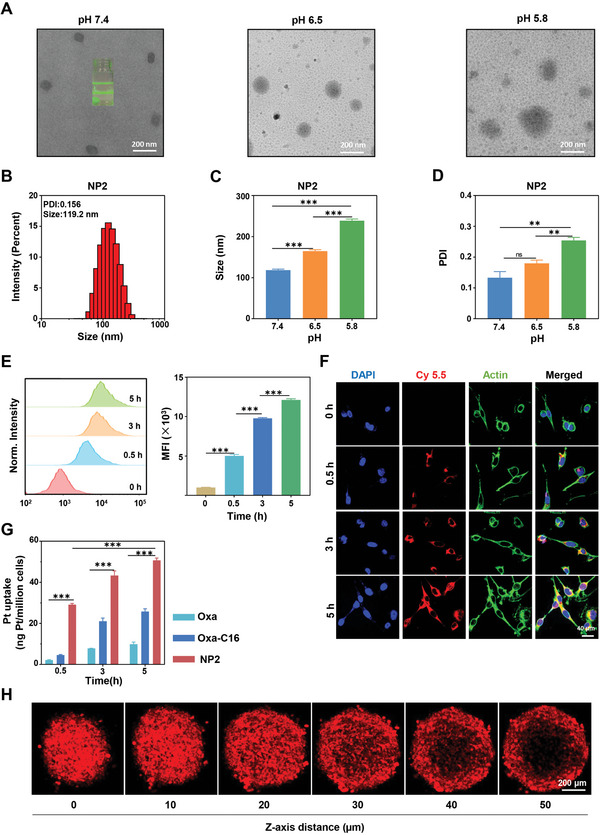
Physical characterization and cellular uptake of **NP2**. A) Transmission electron microscopy image of **NP2** upon incubation at various pH levels. Scale bar = 200 nm. B) Particle size distribution of **NP2** determined by dynamic light scattering. C) Change in hydrodynamic diameter of **NP2** at various pH levels (*n* = 3 independent experiments). Data are means ± standard deviation (SD). *** *p* < 0.001 determined by ordinary one‐way ANOVA and Tukey post‐hoc tests. D) Change in polydispersity of **NP2** at various pH levels (*n* = 3 independent experiments). Data are means ± SD. ns = no statistical difference, ** *p* < 0.01 determined by ordinary one‐way ANOVA and Tukey post‐hoc tests. E) Time‐dependent cellular uptake of **NP2@Cy5.5** in CT26 cells by flow cytometry (*n* = 3 independent experiments). Data are means ± SD. *** *p* < 0.001 determined by ordinary one‐way ANOVA and Tukey post‐hoc tests. F) Time‐dependent cellular uptake of **NP2@Cy5.5** in CT26 cells by confocal laser scanning microscopy. The cell nucleus was stained with 4′,6‐Diamidino‐2‐phenylindol (DAPI) and the cytoskeleton with Alexa‐488. Scale bar = 40 µm. G) Time‐dependent cellular uptake of **NP2** in comparison to **Oxa** and **Oxa‐C16** in CT26 cells by inductively coupled plasma mass spectrometry (*n* = 3 independent experiments). Data are means ± SD. *** *p* < 0.001 determined by ordinary two‐way ANOVA and Tukey post‐hoc tests. H) Penetration of CT26 multicellular tumor spheroids with **NP2@Cy5.5** by z‐stack confocal laser scanning microscopy. Scale bar = 200 µm.

Based on the amphiphilic nature of the polymer, this compound can self‐assemble into nanoparticles by nanoprecipitation as previously described.^[^ ^14^ ^]^ To enhance the aqueous solubility and stability, the PC7A‐based particles were further coated on the surface with a phospholipid. The nanoparticles generated upon functionalization of PC7A with 1,2‐distearoyl‐sn‐glycero‐3‐phosphoethanolamine‐N‐[polyethylene glycol‐2000] ammonium (DSPE‐PEG_2000_) are referred to as **NP1** and the nanoparticles formed upon functionalization of PC7A with DSPE‐PEG_2000_ and encapsulation of **Oxa‐C16** are referred to as **NP2**. Using transmission electron microscopy, the particles were found with a spherical morphology with an average diameter of 71 nm for **NP1** (Figure S4A, Supporting Information) and 105 nm for **NP2** (**Figure** **2**A). Complementary, the hydrodynamic diameter of **NP1** was found to be 81 nm and of **NP2** of 119 nm by dynamic light scattering measurements. The polydispersity of the nanomaterials indicated a uniform distribution of the particles (Figure S4B, Supporting Information and Figure 2B). As a crucial property for a biological application, the stability of the nanomaterials was studied. The nanoparticles were found to be highly stable under physiological conditions (pH = 7.4). Contrarily, the morphology drastically changed and the particle size was strongly augmented (Figure S4A,C,D, Supporting Information and Figure 2B–D) in an acidic environment (pH = 5.8, 6.5). Based on the presence of a tertiary amine moiety in the side chain of the polymer, the zeta potential increased with a reduction of the pH level (Figure S4E,F, Supporting Information). Overall, these findings indicate that the nanoparticles show a strong response to the acidic environment found in the tumor microenvironment.

### 2.2 Cellular Uptake and Cytotoxic Anticancer Effect

To evaluate the cellular uptake of the nanomaterial, **NP2** was labeled with the fluorescent dye Cy5.5 to form the nanoparticle formulation **NP2@Cy5.5**. Colorectal cancer cells (CT26) were incubated with **NP2@Cy5.5** and the internalization into the cells was monitored by flow cytometry. Upon prolongation of the incubation time, increasing amounts of the nanoparticles were found in the cancer cells (Figure 2E). The cellular uptake was further studied by confocal laser scanning microscopy. As expected, with an increasing incubation time, higher amounts of the red fluorescence of **NP2@Cy5.5** were observed inside the cells, suggestive of the augmented cellular uptake (Figure 2F). Complementary, the internalization into the cancer cells of **NP2** in comparison to **Oxa** and **Oxa‐C16** was assessed upon determination of the platinum content inside the cancer cells by inductively coupled plasma mass spectrometry. Promisingly, the nanoparticle formulation showed a significantly higher cellular uptake than the molecular agents (Figure 2G), indicative of the application of the nanoparticles as a drug delivery system. To study the ability of the particles to accumulate in tumorous tissue, the penetration of **NP2@Cy5.5** in CT26 multicellular tumor spheroids was investigated by z‐stack confocal laser scanning microscopy. Promisingly, fluorescence signals stemming from the nanoparticles were found at every section depth, indicating the ability of **NP2@Cy5.5** to fully penetrate 3D cellular architectures (Figure 2H). These findings indicate the high cellular uptake of **NP2** and its ability to penetrate 3D cellular structures.

**Figure 3:**
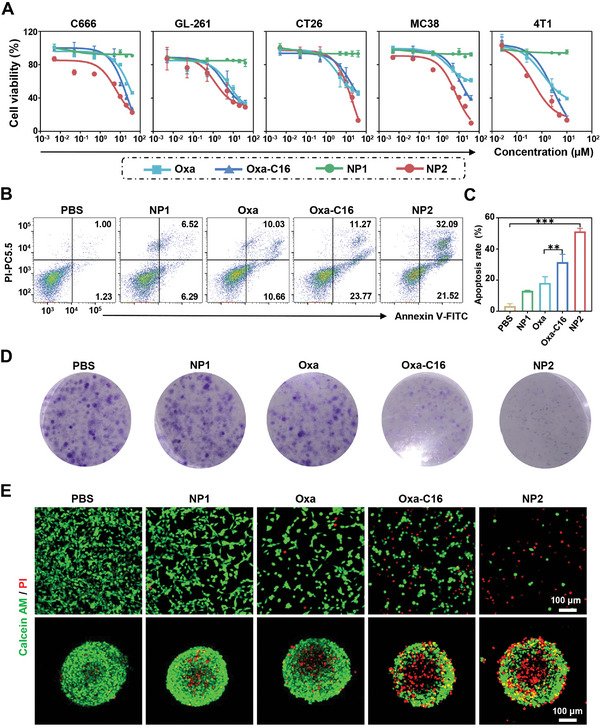
Cytotoxic evaluation of **Oxa**, **Oxa‐C16**, **NP1**, or **NP2** in a cellular and multicellular tumor spheroid model. A) Drug‐response curves against colorectal (CT26 and MC38), breast (4T1), nasopharyngeal carcinoma (C666), and glioma (GL261) cancer cells. B) Evaluation of an apoptotic cell death mechanism upon treatment of CT26 cells and incubation with annexin V/propidium iodide. C) Quantification of the amount of cancer cells in an apoptotic state upon treatment (*n* = 3 independent experiments). Data are means ± SD. ** *p* < 0.01, *** *p* < 0.001 determined by ordinary one‐way ANOVA and Tukey post‐hoc tests. D) Microscopy images of the colony formation of CT26 cells upon treatment. E) Confocal laser scanning microscopy images of CT26 cells (top) or CT26 multicellular tumor spheroids (bottom) upon treatment and incubation with the cell live/dead stain calcein‐AM/propidium iodide. Scale bar = 100 µm.

The cytotoxicity of **Oxa**, **Oxa‐C16**, **NP1**, and **NP2** was assessed against colorectal (CT26 and MC38), breast (4T1), nasopharyngeal carcinoma (C666), and glioma (GL261) cancer cells. The nanoparticle formulation without the therapeutic platinum complex **NP1** was found to be non‐toxic toward all cell lines (IC_50,NP1_ > 100 µm, Figure S5, Supporting Information). The molecular therapeutic platinum complexes showed to cause cell death in the micromolar range (IC_50,OXA_ = 1.09–36.76 µm, IC_50,OXA‐C16_ = 2.89–22.57 µm). Interestingly, the nanoparticle formulation with the platinum complex **NP2** was found to be approximately twice as cytotoxic (IC_50,NP2_ = 0.45–24.06 µm) as the molecular compounds **Oxa** and **Oxa‐C16** (**Figure** **3**A, Table S1, Supporting Information). As a complementary technique for the cytotoxic effect, the treated cancer cells were incubated with the cell live/dead stain calcein‐AM/propidium iodide. While the cell population incubated with **NP1** consisted entirely of living cells, the cancer cells treated with **Oxa** or **Oxa‐C16** showed a mixture of living and dead cells. In comparison, the majority of the cancer cell population treated with **NP2** majorly consisted of dead cells (Figure 3E top panel), confirming the high therapeutic effect of **NP2**. For a deeper understanding of the cell death, the treated cancer cells were incubated with annexin V/propidium iodide and analyzed by flow cytometry. The results showed a significant amount of early and late apoptosis (Figure 3B), suggesting apoptotic processes as the cell death mechanism. A quantification of the treated cell population suggested that approximately twice as much cells were found in an apoptotic state upon treatment with **NP2** as upon treatment with **Oxa** or **Oxa‐C16** (Figure 3C). The influence on the tumor proliferation upon treatment was evaluated in a colony formation assay. While the cancer cells treated with phosphate‐buffered saline (≈80 colonies), **Oxa** (≈40 colonies), or **NP1** (≈80 colonies) were found with high amounts of colonies, the cancer cells treated with **Oxa‐C16** or **NP2** showed negligible colony formation (Figure 3D). To study the therapeutic effect toward 3D cellular architectures, treated multicellular tumor spheroids were incubated with calcein‐AM/propidium iodide. While the multicellular tumor spheroid treated with **NP1** or **Oxa** majorly consisted of living cells, the multicellular tumor spheroid treated with **Oxa‐C16** and in particular with **NP2** showed significant cellular damage as indicated by the large amount of dead cells in the multicellular tumor spheroid (Figure 3E bottom panel). Overall, these results indicate the high therapeutic effect of **NP2** toward various cancer cell lines as well as multicellular tumor spheroids.

### 2.3 Activation of the STING Pathway in Cancer Cells

**Figure 4:**
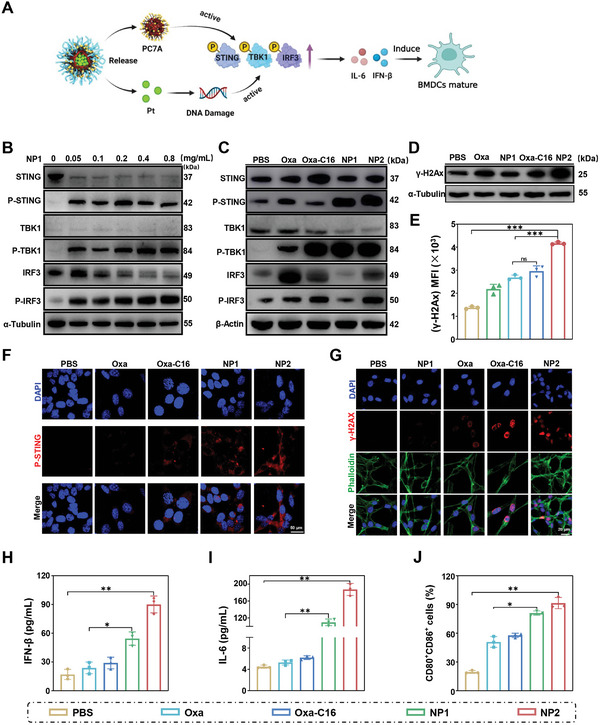
Evaluation of the ability of **Oxa**, **Oxa‐C16**, **NP1**, or **NP2** to intervene in CT26 cancer cells by the STING pathway for therapeutically enhanced combined chemotherapy and immunotherapy. A) Schematic illustration of the mechanism of action of **NP2** to active the STING pathway. B) Change in the expression levels of STING pathway associated proteins upon concentration dependent treatment with **NP1** determined by Western Blot analysis. C) Change in the expression levels of STING pathway associated proteins upon treatment determined by Western Blot analysis. D) Change in the expression levels of *γ*‐H2A upon treatment determined by Western Blot analysis. E) Comparison of the expression levels of *γ*‐H2A determined by flow cytometry. Data were obtained from Figure S6D, Supporting Information (*n* = 3 independent experiments). Data are means ± SD. ns = no statistical difference, *** *p* < 0.001 determined by ordinary one‐way ANOVA and Tukey post‐hoc tests. F) Immunofluorescence confocal laser scanning microscopy images of P‐STING upon treatment. Scale bar = 50 µm. G) Immunofluorescence confocal laser scanning microscopy images of *γ*‐H2AX upon treatment. Scale bar = 20 µm. H) Change of IFN‐*β* levels upon treatment determined by an ELISA assay (*n* = 3 independent experiments). I) Change of IL‐6 levels upon treatment determined by an ELISA assay (*n* = 3 independent experiments). J) Maturation of mouse bone marrow‐derived dendritic cells determined by flow cytometry. Data were obtained from Figure S6G, Supporting Information (*n* = 3 independent experiments). H–J) *n* = 3 independent experiments. Data are means ± SD. * *p* < 0.05, ** *p* < 0.01 determined by ordinary one‐way ANOVA and Tukey post‐hoc tests.

The proposed design of the nanoparticles as therapeutic agents for enhanced chemotherapy and immunotherapy using the STING pathway (**Figure** **4**A) was then evaluated. The influence on the expression levels of STING pathway associated enzymes upon treatment of CT26 cancer cells with **NP1** was studied by Western Blot analysis. In agreement with previous studies on the STING pathway, the protein levels of phospho‐tank binding kinase 1 (P‐TBK1),^[^ ^15^ ^]^ phospho‐interferon regulatory factor 3 (P‐IRF3),^[^ ^16^ ^]^ and P‐STING were upregulated in dependence of the concentration of the treatment with **NP1** (Figure 4B and Figure S6A, Supporting Information).^[^ ^17^ ^]^ These findings suggest that **NP1** is able to activate the STING pathway. A comparison between all here studied compounds revealed that **NP2** demonstrated the strongest STING pathway activation (Figure 4C and Figure S6B, Supporting Information). Using immunofluorescence confocal laser scanning microscopy, the upregulated expression of P‐TBK1 (Figure S6E, Supporting Information), P‐IRF3 (Figure S6F, Supporting Information), and P‐STING (Figure 4F) inside the cancer cells was visualized. Despite having the same polymer backbone, **NP2** demonstrated a stronger activation of the STING pathway than **NP1**, indicating the dual activation of the STING pathway by the polymer backbone and the encapsulated platinum complexes.

The DNA damage caused during the treatment was assessed upon determination of the expression level of the DNA damage marker protein *γ*‐H2A by Western Blot analysis. Upon treatment with **Oxa**, **Oxa‐C16**, or **NP2**, an enhancement of the expression of *γ*‐H2A was observed (Figure 4D and Figure S6C, Supporting Information). The expression of *γ*‐H2A in the cancer cells was quantified by flow cytometry (Figure 4E and Figure S6D, Supporting Information). The comparison between all here studied compounds revealed that **NP2** showed the strongest enhancement of the expression of *γ*‐H2A and therefore the highest amount of DNA damage. Complementary, the expression of *γ*‐H2A in the cancer cells was visualized by confocal laser scanning microscopy (Figure 4G).

Subsequently, the effects of the treatment of CT26 cells on the cytokines interferon beta (IFN‐*β*) and interleukin‐6 (IL‐6) were studied by an Elisa assay. Interestingly, upon treatment with **NP2,** the levels of IFN‐*β* were found to be approximately three times augmented (Figure 4H) and the levels of IL‐6 ≈35‐times enhanced (Figure 4I), indicating the activation of the STING pathway and the release of interferons and inflammatory factors.^[^ ^18^ ^]^ To understand how these pathways influence the immune response, the treated cancer cells were incubated with mouse bone marrow‐derived dendritic cells and their maturation was monitored by flow cytometry. The molecular platinum complexes **Oxa** and **Oxa‐C16** as well as the nanoparticle formulations **NP1** and **NP2** demonstrated to enhance the amount of mature dendritic cells. The comparison showed that **NP2** had the strongest immunogenic effect (Figure 4J and Figure S6G, Supporting Information). Overall, **NP2** demonstrated to efficiently activate the STING pathway by a dual mechanism involving the PC7A polymer and the DNA damage of the platinum complex. The activation of the STING pathway showed to promote the maturation of dendritic cells and therefore generated an enhanced immune response.

### 2.4 Influence on the Metabolism

**Figure 5:**
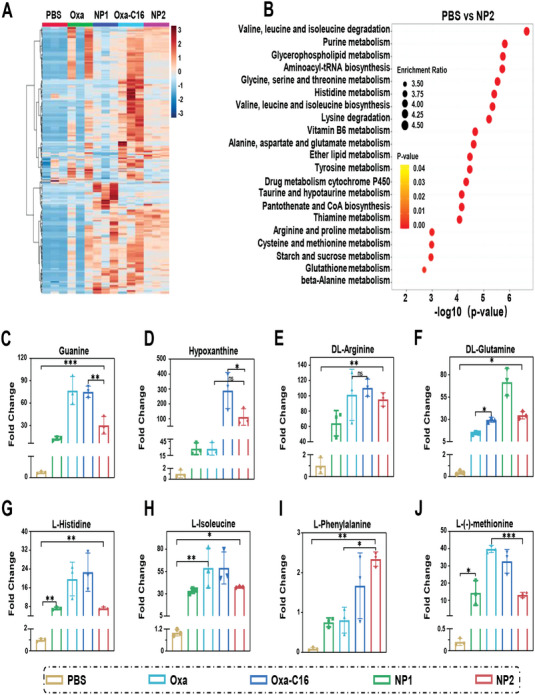
Metabolomics analysis of CT26 cells treated with **Oxa**, **Oxa‐C16**, **NP1**, or **NP2**. A) Heat map of the levels of identified metabolites upon treatment. B) KEGG enrichment analysis of the treatment with **NP2** in comparison to the incubation with phosphate‐buffered saline. The size of the points corresponds to the enrichment ratio and the color of the point corresponds to the relevant p‐value. C,D) Relative change in the respective purine‐based metabolite upon treatment (*n* = 3 independent experiments). Data are means ± SD. ns = no statistical difference, * *p* < 0.05, ** *p* < 0.01, *** *p* < 0.001 determined by ordinary one‐way ANOVA and Tukey post‐hoc tests. E–J) Relative change in the respective amino acid‐based metabolite upon treatment (*n* = 3 independent experiments). Data are means ± SD. ns = no statistical difference, * *p* < 0.05, ** *p* < 0.01, *** *p* < 0.001 determined by ordinary one‐way ANOVA and Tukey post‐hoc tests.

For a deeper insight into the mechanism of action, CT26 cells were treated with **Oxa**, **Oxa‐C16**, **NP1**, or **NP2** and the influence on the cell metabolism was studied by liquid chromatography mass spectrometry. An analysis of the cancer cells revealed in total 412 different metabolites. The change in levels of the respective metabolites is represented in a heat map (**Figure** **5**A). During a KEGG enrichment analysis, specific metabolic pathways which are influenced during the treatment were identified. The molecular therapeutic platinum complex **Oxa** demonstrated to modify the nucleobase, arginine/proline, beta‐Alanine, and glutathione metabolism. These findings are in agreement with previous studies of therapeutic platinum complexes in which this type of compounds demonstrated to coordinate to nucleobases, metal binding amino acids as well as to deplete glutathione in cancerous cells and therefore intervene in the respective metabolisms.^[^ ^19^ ^]^ In comparison, the C7A‐based nanoparticles **NP1** were found to majorly influence the amino acid biosynthesis (i.e., phenylalanine, tyrosine, tryptophane, histidine). Previous studies have indicated that the influence on the amino acid metabolism is strongly related to occurrence and development of tumors.^[^ ^20^ ^]^ Interestingly, the platinum complex encapsulated nanoparticles **NP2** were found with a combined metabolic mechanism of action of **NP1** and **Oxa** (Figure S7, Supporting Information). The results showed that the treatment with **NP2** had a strong effect on the amino acid biosynthesis (i.e., valine, leucine, lysine, glycine, serine, threonine, histidine, lysine, tyrosine, arginine, proline, cysteine) and nucleobase biosynthesis (i.e., purine, pyrimidine) (Figure 5B). Importantly, **NP2** showed a significantly stronger effect on the cell metabolism than **NP1**. For a deeper understanding, specific metabolites to cell death and tumor immunity were further investigated. The levels of guanine and hypoxanthine were augmented upon treatment with platinum containing compounds (Figure 5C,D), indicating influences in the DNA biosynthesis and DNA damage. The biosynthesis of arginine, glutamine, histidine, leucine, phenylalanine, and methionine was up to several order of magnitudes enhanced (Figure 5E–J). Previous studies have indicated that these amino acids as well as others are influencing the development of the tumor immunity.^[^ ^20^ ^]^ For example, numerous studies have confirmed that the cysteine is able to influence antigen presentation and activation of T cells,^[^ ^21^ ^]^ arginine is able to promote the differentiation of T cells to central memory T cells,^[^ ^22^ ^]^ and leucine is able to enhance the proliferation and activation of immune cells by activation of the mTOR pathway.^[^ ^23^ ^]^ Overall, these results indicate that **Oxa** and **Oxa‐C16** are able to influence the nucleobase biosynthesis due to the ability to bind to DNA and **NP1** to modify the amino acid biosynthesis. **NP2** demonstrated to combine both mechanisms of action and therapeutically intervene through the modification of the amino acid and nucleobase biosynthesis, highlighting its use as a multimodal chemoimmunotherapeutic agent.

### 2.5 Biodistribution and Antitumor Activity in Syngeneic Mouse Model

Previous studies have demonstrated that the antitumor effect of chemoimmunotherapeutic agents could be enhanced upon combination with immune checkpoint inhibitors. The chemoimmunotherapeutic treatment generates localized cellular damage as well as releases pro‐inflammatory factors, which promote dendritic cell maturation. To enhance the immunogenic effect, immune checkpoint inhibitors such as the **PD‐L1** monoclonal antibody could bind on the cancer cell surface and diminish the tumor suppressive effect of the cancer cell, resulting in an enhanced immunogenic response.^[^ ^24^ ^]^ Based on these previous investigations, herein, the antitumor effect of **NP2** combined with the **PD‐L1** monoclonal antibody for therapeutically enhanced multimodal chemotherapy and immunotherapy was investigated.

As a crucial requirement for a medicinal application, the biocompatibility of the therapeutic agents was evaluated. 6‐week‐old healthy mice were intravenously injected with phosphate‐buffered saline, **PD‐L1**, **Oxa**, **NP1**, **NP2**, or combined with **NP2+PD‐L1** every 3 days for a period of 15 days, and the body weight of the animals was monitored. No stress, discomfort, or changes in the body weight of the mice were observed in all groups (Figure S8A, Supporting Information). After this time, the mice were sacrificed and the blood as well as major organs were collected and biochemically analyzed. No significant changes in any of the biochemical markers obtained from the liver or kidneys of the animals were observed (Figure S8B–L, Supporting Information). No hemolysis of the blood of the mice was noticed (Figure S9, Supporting Information). The major organs (heart, liver, spleen, lungs, kidneys) of the animals were histologically analyzed by a hematoxylin and eosin stain. No morphological changes in the respective tissues were observed (Figure S10, Supporting Information). Taken together, these results indicate the high biocompatibility of the treatment.

**Figure 6:**
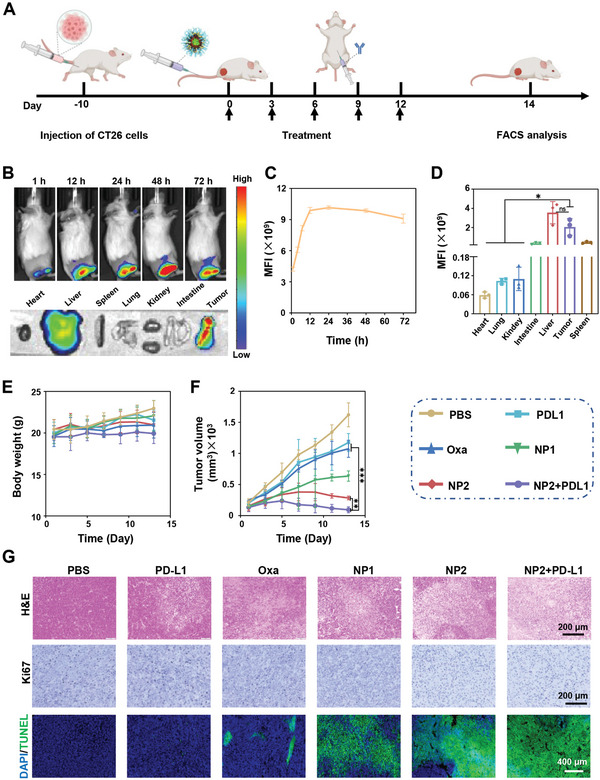
Evaluation of the biological properties of phosphate‐buffered saline, **PD‐L1**, **Oxa**, **NP1**, **NP2**, or **NP2+PD‐L1** in a CT26 tumor syngeneic mouse model. A) Schematic illustration of the timeline of the establishment and treatment of the syngeneic mouse model. B) Fluorescence images upon intravenous injection of **NP2@Cy7.5**. C) Time‐dependent monitoring of the fluorescence upon intravenous injection of **NP2@Cy7.5**. Data obtained from B. D) Biodistribution of **NP2@Cy7.5** in all major organs determined by sacrifice of the animal 72 h after intravenous injection and fluorescence imaging of the organs (*n* = 3 biologically independent mice). Data are means ± SD. ns = no statistical difference, * *p* < 0.05 determined by ordinary one‐way ANOVA and Tukey post‐hoc tests. E) Changes of the body weight upon treatment (*n* = 5 biologically independent mice). F) Tumor growth inhibition curve upon treatment (*n* = 5 biologically independent mice). Data are means ± SD. ** *p* < 0.01, *** *p* < 0.001 determined by ordinary two‐way ANOVA and Tukey post‐hoc tests. G) Hematoxylin and eosin (top, scale bar = 200 µm), Ki67 (middle, scale bar = 200 µm), and terminal deoxynucleotidyl transferase‐mediated dUTP‐biotin nick end labeling (bottom, scale bar = 400 µm) stain of the tumor after the treatment (day 14).

The biodistribution of the fluorescently tagged nanoparticles **NP2@Cy7.5** was studied by fluorescence imaging upon intravenous injection in a CT26 tumor syngeneic mouse model. The images showed that the nanoparticles quickly accumulated in the tumorous tissue (**Figure** **6**B). Notably, 12 h after injection the maximal doses of the nanoparticles was found inside the tumor which gradually decreased upon monitoring for longer periods of time (Figure 6C). 72 h after injection, the mice were sacrificed and the respective organs were harvested. To determine the biodistribution of **NP2** in the mouse model, the fluorescence of the respective organs was detected. As the predominant localization, similar amounts of **NP2@Cy7.5** were observed inside the liver and the tumor (Figure 6D). Overall, these results indicate that the nanoparticles could quickly accumulate at the tumor site.

The therapeutic properties of the chemoimmunotherapeutic agents in combination with the immune checkpoint inhibitor were evaluated toward a CT26 tumor syngeneic mouse model. The chemoimmunotherapeutic agent was intravenously and the immune checkpoint inhibitor intraperitoneal injected every 3 days for a period of 2 weeks. No changes in the weight of the mice were observed during the time frame of the treatment (Figure 6E). The tumor of the mice treated with phosphate‐buffered saline, **PD‐L1**, or **Oxa** grew exponentially. Notably, previous studies have found that the treatment with **PD‐L1** alone could be poorly effective due to poor access to CD8^+^ T cells or immunosuppressive M2 phenotypes in the tumor microenvironment.^[^ ^24^, ^25^ ^]^ In comparison, the treatment with **NP1** and in particular with **NP2** demonstrated a strong tumor growth inhibition effect. The combination treatment of **NP2+PD‐L1** showed to nearly fully eradicate the tumor (Figure 6F). Following the treatment period, all the mice were sacrificed and the tumorous tissue was collected. The enhanced therapeutic effect of the combination treatment of **NP2+PD‐L1** could be rationalized by the multimodal immune stimulation.^[^ ^26^ ^]^ For a deeper understanding of the therapeutic effect, the tumorous tissue was histologically examined. The hematoxylin and eosin and terminal deoxynucleotidyl transferase‐mediated dUTP‐biotin nick end labeling stain showed clear nuclear fragmentation and nuclear lysis of the cancer cells during the treatment with the nanoparticles (Figure 6G, top/bottom panel). The Ki67 stain demonstrated large populations of proliferative cancer cells upon treatment with phosphate‐buffered saline or **PD‐L1**, however, only a few proliferative cells upon treatment with **NP2** or **NP2+PD‐L1** (Figure 6G, middle panel). Overall, these findings demonstrated the strong therapeutic potential of the combination therapy of **NP2+PD‐L1** by multimodal chemotherapy and immunotherapy.

### 2.6 Enhancement of the Anticancer Immune Response in Syngeneic Mouse Model by Activation of the STING Pathway

**Figure 7:**
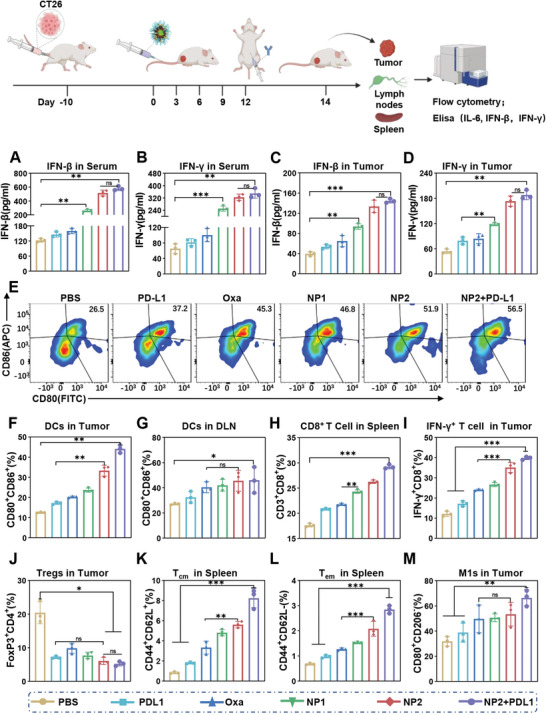
Activation of proinflammatory cytokines or innate immune system in a CT26 tumor syngeneic mouse model upon treatment with phosphate‐buffered saline, **PD‐L1**, **Oxa**, **NP1**, **NP2**, or **NP2+PD‐L1**. A,B) Levels of IFN‐*β* or IFN‐*γ* in the serum upon treatment (*n* = 3 biologically independent animal models). Data are means ± SD. ns = no statistical difference, ** *p* < 0.01, *** *p* < 0.001 determined by ordinary one‐way ANOVA and Tukey post‐hoc tests. C,D) Levels of IFN‐*β* or IFN‐*γ* in the tumor upon treatment (*n* = 3 biologically independent animal models). Data are means ± SD. ns = no statistical difference, ** *p* < 0.01, *** *p* < 0.001 determined by ordinary one‐way ANOVA and Tukey post‐hoc tests. E,F) Levels of CD80^+^ and CD86^+^ in the tumor upon treatment (*n* = 3 biologically independent animal models). Data are means ± SD. ns = no statistical difference, ** *p* < 0.01 determined by ordinary one‐way ANOVA and Tukey post‐hoc tests. G) Levels of CD80^+^ and CD86^+^ in the lymph nodes upon treatment (*n* = 3 biologically independent animal models). Data are means ± SD. ns = no statistical difference, * *p* < 0.05 determined by ordinary one‐way ANOVA and Tukey post‐hoc tests. H) Levels of CD8^+^ in the spleen upon treatment (*n* = 3 biologically independent animal models). Data are means ± SD. ns = no statistical difference, ** *p* < 0.01, *** *p* < 0.001 determined by ordinary one‐way ANOVA and Tukey post‐hoc tests. I) Levels of IFN‐*γ* ^+^ in the tumor upon treatment (*n* = 3 biologically independent animal models). Data are means ± SD. ns = no statistical difference, *** *p* < 0.001 determined by ordinary one‐way ANOVA and Tukey post‐hoc tests. J) Levels of Tregs in the tumor upon treatment (*n* = 3 biologically independent animal models). Data are means ± SD. ns = no statistical difference, * *p* < 0.05 determined by ordinary one‐way ANOVA and Tukey post‐hoc tests. K) Levels of central memory T cells in the spleen upon treatment (*n* = 3 biologically independent animal models). Data are means ± SD. ** *p* < 0.01 *** *p* < 0.001 determined by ordinary one‐way ANOVA and Tukey post‐hoc tests. L) Levels of effector memory T cells in the spleen upon treatment (*n* = 3 biologically independent animal models). Data are means ± SD. *** *p* < 0.01 determined by ordinary one‐way ANOVA and Tukey post‐hoc tests. M) Levels of M1‐type macrophages in the tumor upon treatment (*n* = 3 biologically independent animal models). Data are means ± SD. ns = no statistical difference, ** *p* < 0.01 determined by ordinary one‐way ANOVA and Tukey post‐hoc tests.

Capitalizing on the strong anticancer response inside the CT26 syngeneic mouse model, the immunogenic effect was further evaluated upon biochemical analysis of the blood, tumor, spleen, and lymph tissue of the treated animal models. The levels of the immune indices IFN‐*β*, IFN‐*γ*, and IL‐6 were slightly elevated upon treatment with **NP1** and strongly augmented upon treatment with **NP2** or **NP2+PD‐L1** inside the serum (**Figure** **7**A,B and Figure S11A, Supporting Information) as well as tumorous tissue (Figure 7C,D and Figure S11B, Supporting Information). These results indicate the activation of the STING pathway inside the animal model upon treatment with the nanoparticles.

The maturation of dendritic cells in the tumors and lymph nodes of the treated animal models was studied by flow cytometry. The results showed approximately twice as many mature dendritic cells (CD80^+^ CD86^+^) in the tumor and the lymph nodes upon treatment with **NP2** or **NP2+PD‐L1** than upon treatment with phosphate‐buffered saline, **PD‐L1**, or **Oxa** (Figure 7E–G). Additionally, augmented levels of CD8^+^ in the tumor were observed upon treatment with **NP2** or **NP2+PD‐L1** (Figure 7I and Figure S15, Supporting Information). In agreement with these observations, the levels of CD4^+^ (Figure S11C, Supporting Information) and CD8^+^ (Figure 7H and Figure S14, Supporting Information) T cells in the spleen were enhanced upon treatment with **NP2** or **NP2+PD‐L1**, indicating an immune response within the whole animal model. These results suggest that **NP2** can promote the maturation of dendritic cell and the mature dendritic cells can stimulate T lymphocytes, ultimately activating the immune response.

The levels of type 1 (M1) and type 2 (M2) tumor‐associated macrophages (M1: CD80^+^ CD206^−^; M2: CD80^+^ CD206^+^) upon treatment were studied. The results showed that the treatment with **NP2** or **NP2+PD‐L1** resulted in an enhancement of M1 as well as reduction of M2 (Figure 7M; Figures S11D and S18, Supporting Information), indicating the transformation of M2 to M1.

The accumulation and proliferation of regulatory T cells (Tregs) in the tumor could result in tumor immune escape, which can down‐regulate the expression of CD86 and CD80 on dendritic cells through the surface expression of CTL‐4.^[^ ^27^ ^]^This could result in the inhibition of the maturation of dendritic cells, inhibition of the activation of CD8+ T cells, and therefore hamper the immune response of the organism. To investigate the effect of the treatment on this regulatory mechanism, the level of Tregs in the tumorous tissue was determined. Promisingly, the results showed strongly reduced levels of Tregs (Figure 7J and Figure S16, Supporting Information), indicating the inhibition of immune escape and therefore enhanced therapeutic immunogenic effect.

The ability of the treatment to produce memory cells in the spleen for a long‐lasting immunogenic response was investigated. The results showed strongly enhanced levels of central and effector memory T cells upon treatment with **NP2** or **NP2+PD‐L1** (Figure 7K,L and Figure S17, Supporting Information). Overall, these findings demonstrate that **NP2** and in particular in combination with **PD‐L1** is able to efficiently activate the tumor immune response, reprogram the tumor microenvironment, and induce long‐term immunity against cancer cells.

### 2.7 Statistical Analysis

The statistical analysis of the data was done with the GraphPad Prism 8 software package (GraphPad). The raw data was pre‐processed by transformation or normalization. Data are presented as mean ± standard deviation. All experiments were at least three times repeated (*n* = 3). The level of significance was set at *P* < 0.05. One‐way or two‐way analysis of variance (ANOVA) and Tukey post‐hoc tests were used when more than two or multiple groups were compared. The type of test, number of independent repeats, as well as level of significance is mentioned in the caption of each figure/experiment.

### 2.8 Ethical Approval

All animal experiments were conducted in compliance with the ethical regulations for animal testing and received approval from the Peking University Institutional Animal Care and Use Committee (LA2021316).

## 3 Conclusions

In summary, the encapsulation of chemotherapeutic platinum complexes with the immunomodulating PC7A‐based polymer into pH‐responsive nanoparticles for multimodal therapeutically enhanced chemotherapy and immunotherapy by dual activation of the STING pathway is reported. While the nanomaterial remained stable under physiological conditions, the nanoparticles quickly degraded in the acidic tumor microenvironment, releasing the chemotherapeutic platinum complex as well as the immunogenic C7A monomeric units. This multimodal mechanism allowed for the dual activation of the STING pathway by DNA fragmentation and C7A STING binding. The STING pathway could promote the maturation of dendritic cells and improve the cross‐presentation of dendritic cells, ultimately generating memory T cells for long‐lasting enhanced antitumor immunity. To further enhance the therapeutic profile, these multimodal nanoparticles were combined with an immune checkpoint inhibitor and the biological properties were assessed in a colorectal tumor syngeneic mouse model. This combined treatment demonstrated to fully eradicate the colorectal tumor inside the animal model. Overall, the approach of targeting the STING signaling pathway could present a promising target for novel anticancer agents. The ability of the nanoparticles to intervene through the localized generation DNA damage and the induction of an immune response presents a promising method to prevent or treat tumor metastases as well as tumor reoccurrences.

## Conflict of Interest

The authors declare no conflict of interest.

## Supporting information

Supporting Information

## Data Availability

The data that support the findings of this study are available in the supplementary material of this article.
