# Supplementary material for: Encapsulation of Platinum Prodrugs into PC7A Polymeric Nanoparticles Combined with Immune Checkpoint Inhibitors for Therapeutically Enhanced Multimodal Chemotherapy and Immunotherapy by Activation of the STING Pathway
Source: Adv Sci (Weinh). 2022 Dec 11;10(4):2205241. doi: 10.1002/advs.202205241 (PMC9896041; doi:10.1002/advs.202205241)
Supplement: Supplementary file 1 — Supporting Information [file ADVS-10-2205241-s001.pdf]

ADVANCED SCIENCE
Open Access

Supporting Information

for *Adv. Sci.*, DOI 10.1002/advs.202205241

Encapsulation of Platinum Prodrugs into PC7A Polymeric Nanoparticles Combined with Immune Checkpoint Inhibitors for Therapeutically Enhanced Multimodal Chemotherapy and Immunotherapy by Activation of the STING Pathway

Xiangjie Gao, Guanxiong Lei, Bin Wang, Zhong Deng, Johannes Karges, Haihua Xiao* and Donghui Tan**

Supporting Information

Encapsulation of Platinum Prodrugs into PC7A Polymeric Nanoparticles Combined with Immune Checkpoint Inhibitors for Therapeutically Enhanced Multimodal Chemotherapy and Immunotherapy by Activation of the STING Pathway

Xiangjie Gao[1,2,3,4,#], Guanxiong Lei[1,2,3,4,#], Bin Wang[5], Zhong Deng[1,2,3,4], Johannes Karges[6,*], Haihua Xiao[5,*], Donghui Tan[1,2,3,4,*]

[1]Affiliated Hospital of Xiangnan University, Chenzhou, Hunan Province, 423000, China

[2]Xiangnan University, Chenzhou, Hunan Province, 423000, China

[3]Key Laboratory of Medical Imaging and Artificial Intelligence of Hunan Province, Chenzhou, Hunan Province, 423000, China

[4]Hunan Engineering Research Center of Advanced Embedded Computing and Intelligent Medical Systems, Xiangnan University, Chenzhou, Hunan Province, 423000, China.

[5]Beijing National Laboratory for Molecular Sciences, State Key Laboratory of Polymer Physics and Chemistry, Institute of Chemistry, Chinese Academy of Sciences, Beijing, 100190, China.

[6]Faculty of Chemistry and Biochemistry, Ruhr-University Bochum, Universitätsstrasse 150, 44780, Bochum, Germany

Experimental Section

Materials

All chemicals were obtained from commercial sources and were used without further purification. 2,2,5-Trimethyl-1,3-diOxane-5-carboxylic acid, 2-(azepan-1-yl)ethanol, 1-ethyl-3-(3-dimethylaminopropyl)carbodiimide (EDC·HCl), 4-(dimethylamino)pyridine (DMAP), 2,2,5-Trimethyl-1,3-diOxane-5-carboxylic acid (TDC), N-(2-Hydroxyethyl)hexamethyleneimine (NHL), trifluoroacetic acid (TFA), ethyl-2,6-diisocyanatohexanoate, $mPEG_{5000}$-OH, DSPE-PEG_{2000}, and 3-(4,5-dimethylthiazol-2-yl)-2,5-diphenyltetrazolium bromide (MTT) were purchased from Aladdin (Shanghai, China). Oxaliplatin (Oxa) was purchased from Shandong Boyuan Chemical Company (Shandong, China). Cell media, penicillin/streptomycin (P/S), 0.25% trypsin-EDTA, and fetal bovine serum (FBS) were purchased from Gibco (Gran Island, NY, U.S.A.).

Characterization

^{1}H- and ^{13}C-NMR spectra were recorded on a 400 MHz NMR spectrometer (Bruker). GPC measurements were performed on a shimadzu LC-20A apparatus. Inductively coupled plasma mass spectrometry measurements were recorded on a 7700 inductively coupled plasma spectrometer (Agilent, USA). Transmission electron microscopy images were recorded on a HT-7700 transmission electron microscope (Hitachi, Japan). Dynamic light scattering measurements were performed on a Malvern Zetasizer Nano ZS90 laser particle size analyzer (Nano ZS, UK). Flow cytometry analysis was performed on a CytoFLEX Flow Cytometer instrument (Becton Dickinson, San Jose, CA, USA). Confocal laser scanning microscopy images were recorded on a LSM-880 microscope (ZEISS, Germany).

Synthesis of C7A-1

2,2,5-Trimethyl-1,3-diOxane-5-carboxylic acid (10 mmol, 1.74 g), 2-(azepan-1-yl)ethanol (12 mmol, 1.72 g), 1-ethyl-3-(3-dimethylaminopropyl)carbodiimide (EDC·HCl, 12 mmol, 2.30 g), and 4-(dimethylamino)pyridine (DMAP, 12 mmol, 1.22 g) were dissolved in dichloromethane (20 mL). The mixture was stirred at room temperature for 48 h. The crude product was purified by column chromatography on silica gel using dichloromethane/methanol

(15/1, v/v). The fractions containing the product were combined and the compound dried. Yield: 1.24 g (71%). ^{1}H NMR (400 MHz, $CDCl_3$): δ ppm 4.25-4.17 (m, 4H), 3.65-3.62 (d, 2H), 2.80-2.77 (m, 2H), 2.71-2.68 (q, 4H), 1.63-1.58 (m, 8H), 1.43-1.39 (d, 6H), 1.22 (s, 3H). ^{13}C NMR (400 MHz, $CDCl_3$): δ ppm 176.16, 98.05, 66.00, 63.08, 56.21, 55.66, 41.75, 28.40, 27.00, 23.89, 23.45, 18.78.

Synthesis of C7A-2

C7A1 (1.15g, 3.86mmol) was dissolved in dichloromethane (20 mL), and trifluoroacetic acid (TFA, 0.88g, 7.72mmol) was added dropwise. The mixture was stirred at room temperature overnight. The crude product was purified by column chromatography on silica gel using dichloromethane/methanol (10/1, v/v). The fractions containing the product were combined and the compound dried. Yield: 0.52 g (52%). ^{1}H NMR (400 MHz, $CDCl_3$): δ ppm 4.38-4.34 (t, 2H), 3.73 (s, 4H), 2.76-2.73 (t, 2H), 2.69-2.66 (t, 4H), 1.66-1.61 (q, 8H), 1.14 (s, 3H).

Synthesis of PC7A

C7A-2 (0.2 mmol, 51.84 mg) and ethyl-2,6-diisocyanatohexanoate (0.2 mmol, 47.48 mg) were dissolved in anhydrous dimethylformamide (10 mL). The mixture was stirred at room temperature for 12 h. After this time, $mPEG_{5000}$-OH (0.02 mmol, 1.52 mg) was added and the mixture was heated at 50 °C for 12 h. Distilled water (10 mL) was added and the solution was placed into a dialysis bag. The polymer was dialyzed against distilled water for 48 h. After this time, the polymer was obtained by lyophilization.

Synthesis of Oxa-C16

The compound was synthesized according to a previously reported protocol[1]. Pt(IV)-COOH (520 mg, 0.98 mmol) and hexadecylcarbamic acid (268 mg, 1.0 mmol) were suspended in anhydrous dimethylformamide (10 mL). The mixture was heated at 75 °C overnight. The solvent was removed under reduced pressure. The crude product was purified by recrystallization from methanol. The solid was collected by gravity filtration and the compound dried. ^{1}H NMR (400 MHz, DMSO-d_6): δ ppm 12.06 (s, 1H), 9.58 (s, 1H), 8.66 (s, 1H), 8.18 (s, 2H), 6.73 (s, 1H), 6.28 (s, 1H), 2.89 (s, 2H), 2.58 (s, 2H), 2.40-2.38 (d, 2H), 2.15-2.12 (d, 2H), 1.50 (d, 2H), 1.33-1.24 (d, 34H), 0.87-0.84 (t, 3H).

Preparation of NP1

PC7A (20 mg) and DSPE-PEG$_{2000}$ (10 mg) were dissolved in 200 μL dimethyl sulfoxide (DMSO). The mixed solution was then dropwise added into distilled water (10 mL), which was then exposed to ultrasound for the self-assembly of the nanoparticles. The nanoparticles were dialyzed against distilled water for 24 h, which then obtained NP1.

Preparation of NP2

PC7A (40 mg), Oxa-C16 (6 mg), and DSPE-PEG$_{2000}$ (20 mg) were dissolved in DMSO (600 μL). The solution was then dropwise added into distilled water (10 mL), which was exposed to ultrasound radiation to initiate the self-assembly of the NP2. The NP2 were dialyzed against water for 24 h. The loading of the platinum complex was assessed by inductively coupled plasma mass spectrometry.

Preparation of NP2@Cy5.5 and NP2@Cy7.5

The preparation method was same as NP2. PC7A (40 mg), Cy5.5 (1 mg), Oxa-C16 (6 mg) and DSPE-PEG$_{2000}$ (20 mg) were dissolved in 1 mL DMSO. The obtained DMSO solution was then added into 10 mL water with magnetic stirring to form nanoparticles. Finally, the NP2@Cy5.5 were obtained after ultrafiltration and dialysis. The NP2@Cy7.5 were prepared with the same procedure.

Cell culture

The CT26, MC38, 4T1, C666, and GL261 cells were cultured in RPMI 1640 medium. The cell media was complemented with 10% of FBS and 100 U/mL penicillin-streptomycin mixture. The cells were cultivated in a humidified atmosphere at 37 °C containing 5% CO_2.

Cellular uptake determined by flow cytometry

CT26 cells were seeded into 24-well plates at a density of 5×10^4 cells per well. After 24 h, the cells were incubated with **NP2@Cy5.5** (5 μM Pt) for various periods of time (0.5, 3, and 5 h). The cellular uptake was assessed by flow cytometry.

Cellular uptake determined by confocal laser scanning microscopy

CT26 cells were seeded into 24-well plates at a density of 1 × 10^5 cells per well. After 24 h, the cells were incubated with **NP2@Cy5.5** (5 μM Pt) for various time periods (0.5, 3, and 5 h). The cells were washed with phosphate-buffered saline and then fixed with paraformaldehyde. The cell nucleus was then stained with DAPI, and the cytoskeleton was stained with Alexa-488. In the end, images were taken with confocal laser scanning microscopy.

Cellular uptake determined by inductively coupled plasma mass spectrometry

CT26 cells were seeded into 24-well plates at a density of 5 × 10^4 cells per well. After 24 h, the media was removed and the cells were incubated with Oxa, Oxa-C16, and **NP2** at a Pt concentration of 5 μM for various time periods (0.5, 3, and 5 h). The cells were trypsinised, harvested, centrifuged, and resuspended. The number of cells on the dish was counted. Subsequently, the sample was digested using a 60% nitric acid solution for three days, and then diluted with 2% nitric acid solution. The platinum content was determined by inductively coupled plasma mass spectrometry.

Generation of multicellular tumor spheroids

Agarose solution (1%, W/V, 50 μL) was added to each well of a 96-well plate. UV irradiation was applied on the plate for 30 minutes for sterilization. Subsequently, a cell suspension with 3 × 10^3 cells was seeded on top of the agarose. After 2-3 days, multicellular tumor spheroids were formed. The multicellular tumor spheroids were cultured in a cell culture incubator at 37 °C with 5% CO_2.

***In vitro* Cytotoxicity**

The cytotoxicity of the cancer cells was assessed using a (3-(4,5-dimethylthiazol-2-yl)-2,5-diphenyltetrazolium bromide) (MTT) colorimetric assay. The cells were seeded on 96-well plates (Thermo Scientific, USA) at a density of 6 × 10^3 cells per well. After incubation for 24 h, the cells were incubated with increasing concentrations of Oxa, Oxa-C16, **NP1**, and **NP2** for 48 h. Then, the media was replaced with fresh media containing MTT (10 μL of a 5 mg/mL

solution in phosphate-buffered saline) and the cells were further incubated for 4 h. Acidified SDS solution was then added (100 μL/well) and the plates were kept in the dark for an additional 12 h. Measurements of absorbance were subsequently performed on a Bio-Rad plate reader at 570 nm (peak absorbance) and at 650 nm (background absorbance).

Cell apoptosis assay

CT26 cells were seeded on 6-well plates at a density of 1×10^6 cells per well and cultured overnight. The cells were divided into 6 groups for the cytotoxicity study: 1) phosphate-buffered saline, 2) NP1, 3) Oxa, 4) Oxa-C16, and 5) NP2. Group 3, Group 4, and Group 5 were treated with the indicated drugs at a Pt concentration of 23.25 μM, and Group 2 was treated with polymer concentration at 50 μg/mL. After incubation for 48 h, the cells were washed with phosphate-buffered saline, and then stained with Annexin V/Propidium iodide for 15 min. Finally, the cell apoptosis was detected by flow cytometry, and the data were analyzed by FlowJo software.

Colony formation

CT26 cells were seeded on 6-well plates at a density of 1×10^3 cells per well and cultured overnight. The cells were divided into 6 groups for the cytotoxicity study: 1) phosphate-buffered saline, 2) NP1, 3) Oxa, 4) Oxa-C16, and 5) NP2. Group 3, Group 4, and Group 5 were treated with the indicated drugs at a Pt concentration of 23.25 μM, and Group 2 was treated with polymer concentration at 50 μg/mL. The culture media was replaced every two days. After ten days, the cells were fixed with 4% paraformaldehyde and further incubated with 1% crystal violet (Solabio). The colony formation was monitored by optical microscopy.

Live/dead cell staining

CT26 cells were seeded into 6-well plates at a density of 8×10^4 cells per well and cultured overnight. The cells were divided into 6 groups for the cytotoxicity study: 1) phosphate-buffered saline, 2) NP1, 3) Oxa, 4) Oxa-C16, and 5) NP2. Group 3, Group 4, and Group 5 were treated with the indicated drugs at a Pt concentration of 23.25 μM, and Group 2 was treated

with polymer concentration at 50 μg/mL. After 24 h treatment, the media was removed. The cells were washed with phosphate-buffered saline and then stained with Cell Viability/Cytotoxicity Assay Kit.

Western blot

The CT26 cells were seeded on 6-well plates at a density of 1 × 10^6 cells per well. The cells were divided into 5 groups for WB assays:1) PBS, 2) Oxa, 3) Oxa-C16, 4) **NP1**, 5) **NP2**. Group 2, Group 3, and Group 5 were treated with the indicated drugs at a Pt concentration of 23.25 μM, and Group 4 was treated with polymer concentration at 50 μg/mL. After incubation for 48 h, RIPA lysis buffer (P0013B, Beyotime) with protease and phosphatase inhibitors (P1097, Beyotime) was added into the wells. The proteins were extracted by centrifugation at 12000 rpm for 15 min. The concentration of the proteins was determined using a BCA protein assay kit (P0011, Beyotime). Proteins in equivalent amounts were then separated on a 10% sodium dodecyl sulfate-polyacrylamide gel electrophoresis (SDS-PAGE) and transferred to the PVDF membrane by a gel-electrophoretic apparatus (Bio-Rad mini, USA), followed by blocking in TBS-T solution containing 5% skim milk for 1 h. Subsequently, the membrane was incubated with primary antibody against P53 (#30313, CST), γ-H2AX (#9718, CST), STING (#13647, CST), P-STING (#729711, CST), TBK1 (#38066, CST), P-TBK1 (#5483, CST), IRF3 (#4302, CST), and P-IRF3 (#29047, CST) overnight on a shaker at 4 °C. Subsequently, the PVDF films were washed 3 times and incubated with HRP (A0208, Beyotime) conjugated antibodies for 2 h at room temperature. The Western blot images were obtained by Gel imaging system (Tanon 4800, China) with 200 μL of ECL chemiluminescent reagent (KF001, Affinity) added on the top of the membrane. β-Actin (#AF5003, Beyotime) and α-Tubulin (#9099, CST) were employed as protein loading control.

Immunofluorescence characterization using confocal laser scanning microscopy

The cells were seeded onto cover slides at a density of 1 × 10^5 cells per slide and cultured overnight. The cells were divided into 5 groups for immunofluorescence imaging: 1) PBS, 2) Oxa, 3) Oxa-C16, 4) NP1, 5) NP2. Group 2, Group 3, and Group 5 were treated with the indicated drugs at a Pt concentration of 23.25 μM, and Group 4 was treated with polymer concentration at 50 μg/mL. Then, the cells were fixed in a 4% paraformaldehyde solution,

blocked with 1% BSA (Beyotime) and incubated with 0.1% Triton (Beyotime). Afterwards, the cells were incubated with P-STING, P-TBK1, P-IRF3, and γ-H2A primary antibody diluted in cell media at 4 °C for 12 h. Subsequently, the media was removed and the cells were washed with phosphate-buffered saline. The cells were further incubated with the secondary Alexa Fluor 488-conjugated antibody (ab150077, Abcam) for 2 h. The cell nucleus was stained with DAPI and the cytoskeleton was stained with Phalloidin (Solabio). Images were taken with a confocal laser scanning microscope.

DNA damage evaluation using flow cytometry

The DNA damage in the cancer cells was detected upon monitoring of the DNA damage marker protein γ-H2A. CT26 cells were seeded on 12-well plates at a density of 3×10^5 cells per well and cultured overnight. The cells were divided into 5 groups for immunofluorescence imaging: 1) PBS, 2) Oxa, 3) Oxa-C16, 4) NP1, 5) NP2. Group 2, Group 3, and Group 5 were treated with the indicated drugs at a Pt concentration of 23.25 μM, and Group 4 was treated with polymer concentration at 50 μg/mL. After 48 h of incubation, all the cells were fixed in a 4% paraformaldehyde. Afterwards, the cells were incubated with the γ-H2A primary antibody diluted in cell media at 4 °C for 12 h. Subsequently, the media was removed and the cells were washed with phosphate-buffered saline. The cells were further incubated with the secondary Alexa Fluor 488-conjugated antibody (ab150077, Abcam) for 2 h. The expression of γ-H2A was then detected via flow cytometry.

ELISA assay

CT26 cells were seeded on 12-well plates at a density of 3×10^5 cells per well and cultured overnight. The cells were divided into 5 groups for ELISA: 1) PBS, 2) Oxa, 3) Oxa-C16, 4) NP1, 5) NP2. Group 2, Group 3, and Group 5 were treated with the indicated drugs at a Pt concentration of 23.25 μM, and Group 4 was treated with polymer concentration at 50 μg/mL. Subsequently, the media was removed and the cells were washed with phosphate-buffered saline. The cells were centrifuged at 12000 rpm/min for 5 min. The obtained supernatant was then detected using a mouse IL-6 ELISA kit (EK2236-01, Multi Sciences) and a mouse IFN-β ELISA kit (EK206HS-02, Multi Sciences) according to the manufacturers protocol.

Maturation of bone-marrow derived dendritic cells

Bone-marrow derived dendritic cells were obtained from 5 to 6 week-old female C57BL/6 mice and cultured in RPMI 1640 medium supplement with 10% FBS, granulocyte-macrophage colony-stimulating factor (GM-CSF) (20 ng/mL, Beyond), and interleukin-4 (IL-4) (10 ng/mL, Beyond) at 37 °C with 5% (v/v) CO_2.

CT26 cells were seeded on 12-well plates at a density of 3×10^5 cells per well. After 24 h, the cells were treated with Oxa, Oxa-C16, **NP1**, or **NP2** for 48 h. The CT26 cells were incubated with the obtained bone-marrow derived dendritic cells for 24 h. Subsequently, the dendritic cells were stained with the corresponding antibody (anti-CD11c-PE, anti-CD80-FITC, and anti-CD86-APC) for 1 h. The maturation of the dendritic cells was assessed by flow cytometry.

Metabolomics assay

The cells were seeded on the cell culture plates and cultured overnight. After 24 h, the media was removed and the cells were treated with Oxa, Oxa-C16, **NP1**, or **NP2** for 48 h. For Pt containing group, the concentration for the treatment was fixed at 23.25 μM. For NP1, the concentration for the polymer was 50 μg/mL. Then, the cells were washed with phosphate-buffered saline. Subsequently, the cells were incubated with ice-cold extraction solvent (water: methanol: chloroform = 100 μl : 180 μl : 120 μl) for 1 min. The mixture was centrifuged at 1000 g for 15 minutes at 4 °C. The supernatant was analyzed by UPLC (Ultimate 3000, Thermo Fisher Scientific, San Jose, CA, USA)-ESI-Qrbitrap-MS (Orbitrap Fusion Lumos, Thermo Fisher Scientific, San Jose, CA, USA). Identification and relative quantification of the data were conducted by Compound discoverer (3.1). The follow-up statistical and enrichment analysis of metabolomic and lipidomic was based on MetaboAnalyst 5.0 (https://www.metaboanalyst.ca/).

***In vivo* biocompatibility evaluation**

For the biocompatibility study, healthy Kunming mice were randomly divided into 6 groups: 1) PBS, 2) Oxa, 3) NP1, 4) anti PD-L1, 5) NP2, and 6) NP2 + anti PD-L1. Group 2, Group 5, and Group 6 were intravenously injected with drugs at a Pt dose of 3 mg/kg. Group 4 and Group 6 were intraperitoneal injected with anti PD-L1 at a dose of 5 mg/kg. The body weight of mice was monitored during 15 days treatment. Finally, the major organs were

collected and a slice of each one was fixed with 4% paraformaldehyde. The obtained slices were stained with hematoxylin and eosin.

Hemolysis study

For the hemolysis study, blood samples were obtained from the mice. The blood was centrifuged at 200 rpm for 5 min. The obtained blood serum was incubated with PBS, Oxa, Oxa-C16, **NP1**, **NP2** at 37 °C for 3 h. For Oxa, Oxa-C16, NP2, the samples were treated with the indicated drugs at a Pt concentration of 300 μM. For NP1, the serum was treated with polymer concentration at 500 μg/mL. The absorption at 541 nm was measured with a microplate reader. The hemolysis ratio was calculated using the following formula:

$$HP(\%) = (DT\text{-}DNC) / (DPC\text{-}DN \times 100)$$

DT-experimental group, DNC-negative control group, DPC-positive control group

Biodistribution study

The CT26 tumor bearing mice were administrated with **NP2@Cy7.5** *via* intravenously injection. The fluorescence imaging proceeded using an IVIS system (Spectrum CT, PerkinElmer) at various time intervals. The *in vivo* biodistribution of **NP2@Cy7.5** was observed on the Cy7.5 channel (λ_{ex}= 745 nm, λ_{em}= 840 nm). At 72 h-post injection, the mice were sacrificed, and their organs were harvested and imaged *ex vivo* using the same parameters. The average photon flux in radians for the different reporter signals in each excised organ were quantified.

Antitumor efficacy study

CT26 cells (3×10^6) were subcutaneously injected into the right hip of female BALB/c mice. When the size of tumor reached 100 mm^3, the mice were randomly divided into 6 groups (5 mice in each group), which includes: 1) PBS, 2) Oxa, 3) NP1, 4) anti PD-L1, 5) NP2, and 6) NP2 + anti PD-L1. Group 2, Group 5, and Group 6 were intravenously injected with drugs at a Pt dose of 3 mg/kg. Group 4 and Group 6 were intraperitoneal injected with anti PD-L1 at a dose of 5 mg/kg. The body weight and tumor volume were recorded every two days. The tumor

volume was calculated using the following equation: Tumor volume = $1/2 \times L \times W^2$, where "L" is the long diameter of the tumor, and "W" is the short diameter of the tumor. Data are presented as means ± SD (n =5).

Maturation of dendritic cells in CT26 xenograft mouse model

The peripheral blood was collected from mice treated with different drugs for ELISA tests through centrifuging at a speed of 12000 rpm/min for 5 min. The animal tissues were accurately weighed and 9 times the volume of homogenizing medium (0.86% or 0.9% normal saline is recommended) was added at the ratio of weight (mg) : volume (μl) = 1:9. Under the condition of ice water bath, the homogenization was performed mechanically to prepare 10% homogenate, and the supernatant was centrifuged for 10 minutes from 2500 to 3000 rpm. The above peripheral blood was prepared for cytokines analysis using Mouse IL-6 High Sensitivity ELISA kits, Mouse IFN-β ELISA Kit and Mouse IFN-γ High Sensitivity ELISA kits (EK280HS-01, Multi Sciences).

Analysis of the tumor immune microenvironment in CT26 xenograft mouse model

Fresh tumors, spleen, and draining lymph node tissue were collected for antitumor immune response analysis *via* FACS. Briefly, samples were dissociated into single-cell suspensions, and then red blood cells were removed with red blood cell lysing buffer (Solabio). After that, samples were blocked with 0.1% BSA in PBS followed by incubation with relevant antibodies for 1 h at room temperature. To characterize T cells and TEM in tumors and spleen, cells were stained with anti-mouse CD3-PE, anti-mouse CD4-APC, anti-mouse CD8-FITC, anti-mouse CD44-PC5.5, and anti-mouse CD62L-APC (Biolegend, USA). To analyze DC in tumors and lymph nodes, cells were stained with anti-mouse CD11C-PE, anti-mouse CD80-FITC, and anti-mouse CD86-APC (Biolegend, USA). In order to analyze TAMs (tumor-associated macrophages), Tregs (regulatory T cells), CTL (cytotoxic T cells), and IFN-γ in tumor tissues, anti-mouse F4/F80-PE, anti-mouse CD8-FITC, anti-mouse CD206-APC, anti-mouse CD3-PE, anti-mouse CD4-APC, cells were stained for anti-mouse FOXP3-FITC, anti-mouse CD4-PC5.5, and anti-mouse IFN-γ-APC (Biolegend, USA). Flow cytometry data were collected using CytExpert software and FlowJo software was used to process the data. Figure S12-18 shows the gating policy.

Supplementary Figures

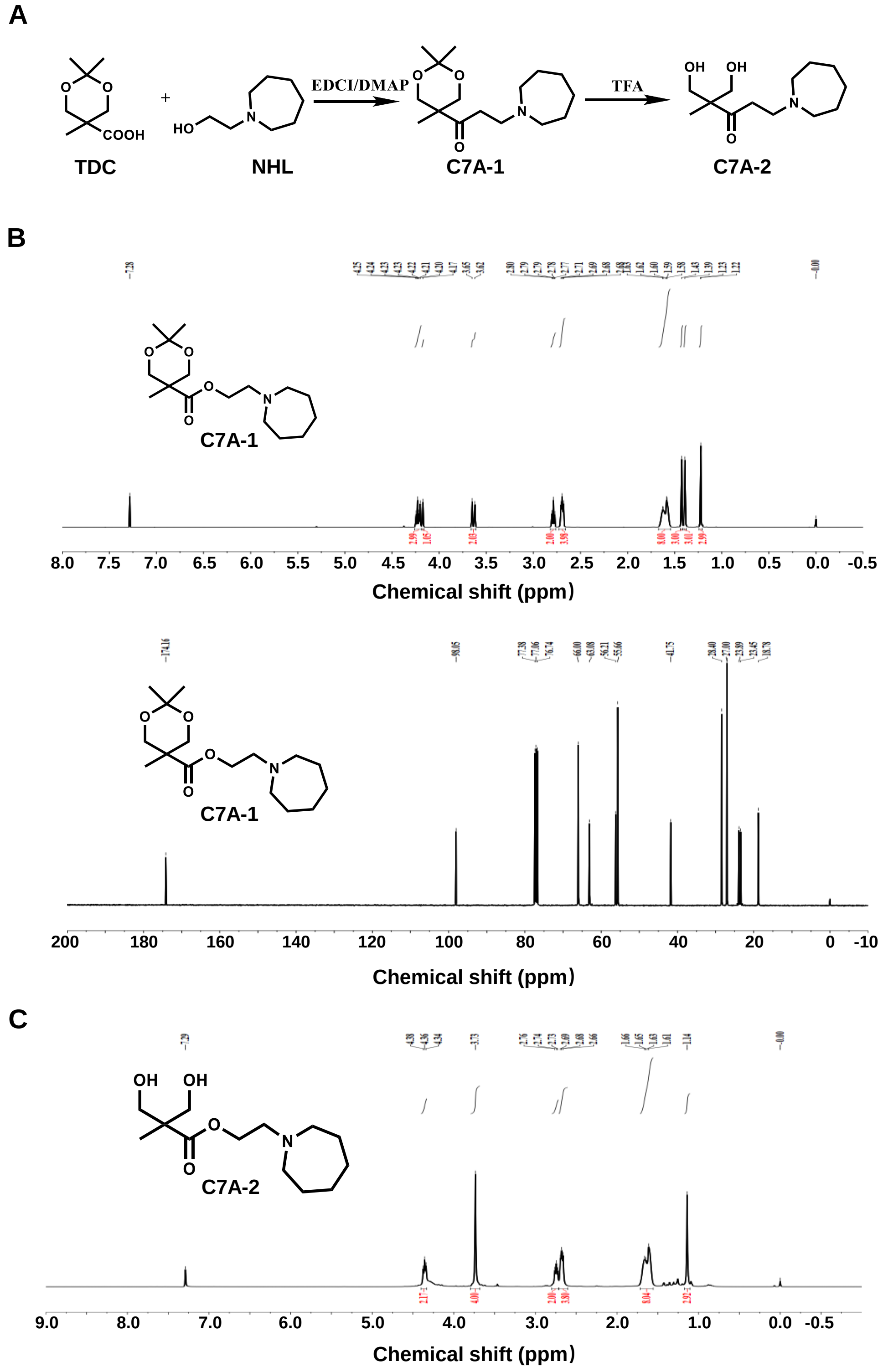

Figure S1. Synthesis and characterization of C7A. (A) Synthesis route for C7A-1 and C7A-2. (B) ^{1}H- and ^{13}C-NMR spectra of C7A-1. (C) ^{1}H-NMR spectrum of C7A-2.

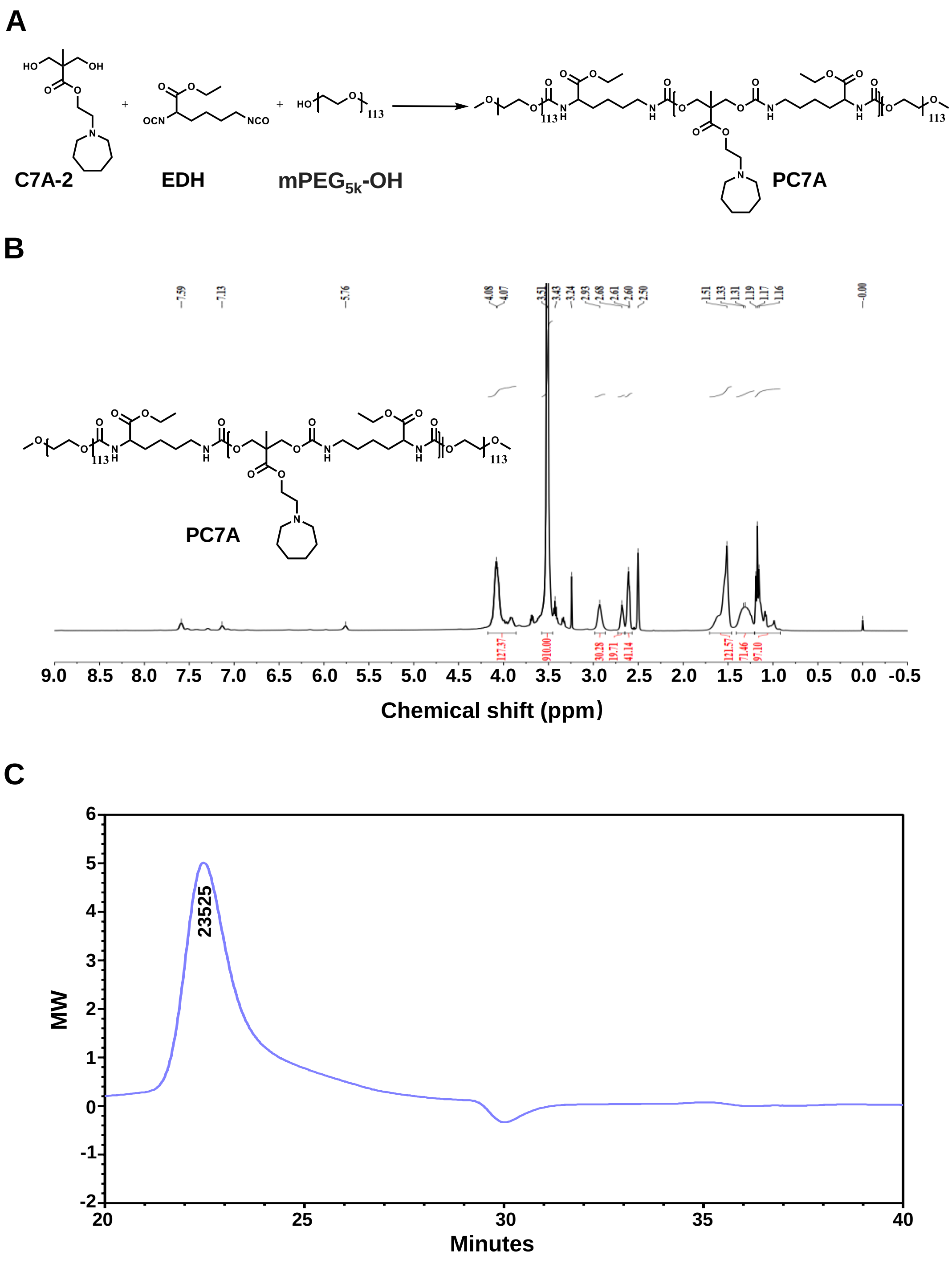

Figure S2. Synthesis and characterization of PC7A. (A) Synthesis route for PC7A. **(**B) ^{1}H-NMR spectrum. (C) Gel permeation chromatogram characterization of PC7A.

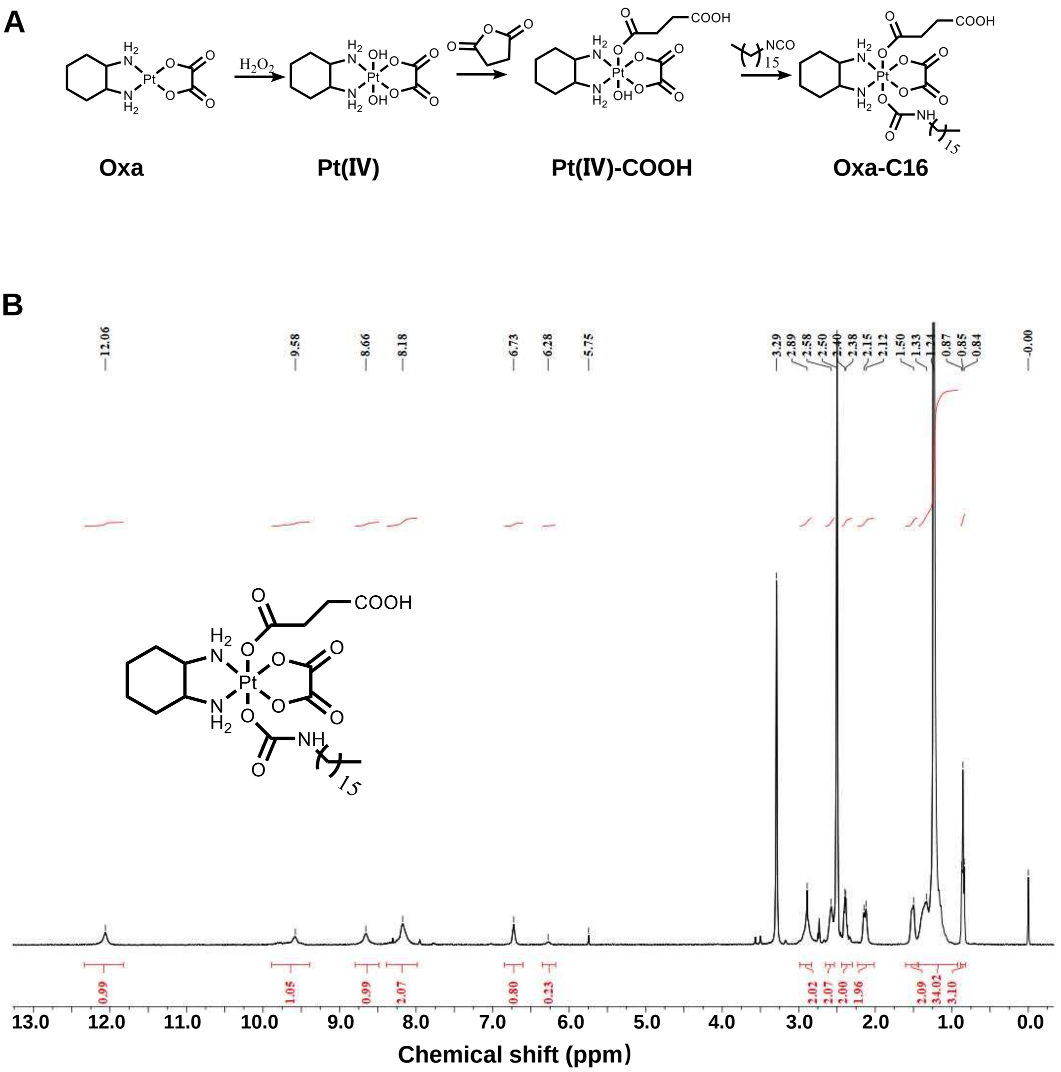

Figure S3. (A) Synthesis route for Oxa-C16. (B) ^{1}H-NMR spectrum of Oxa-C16.

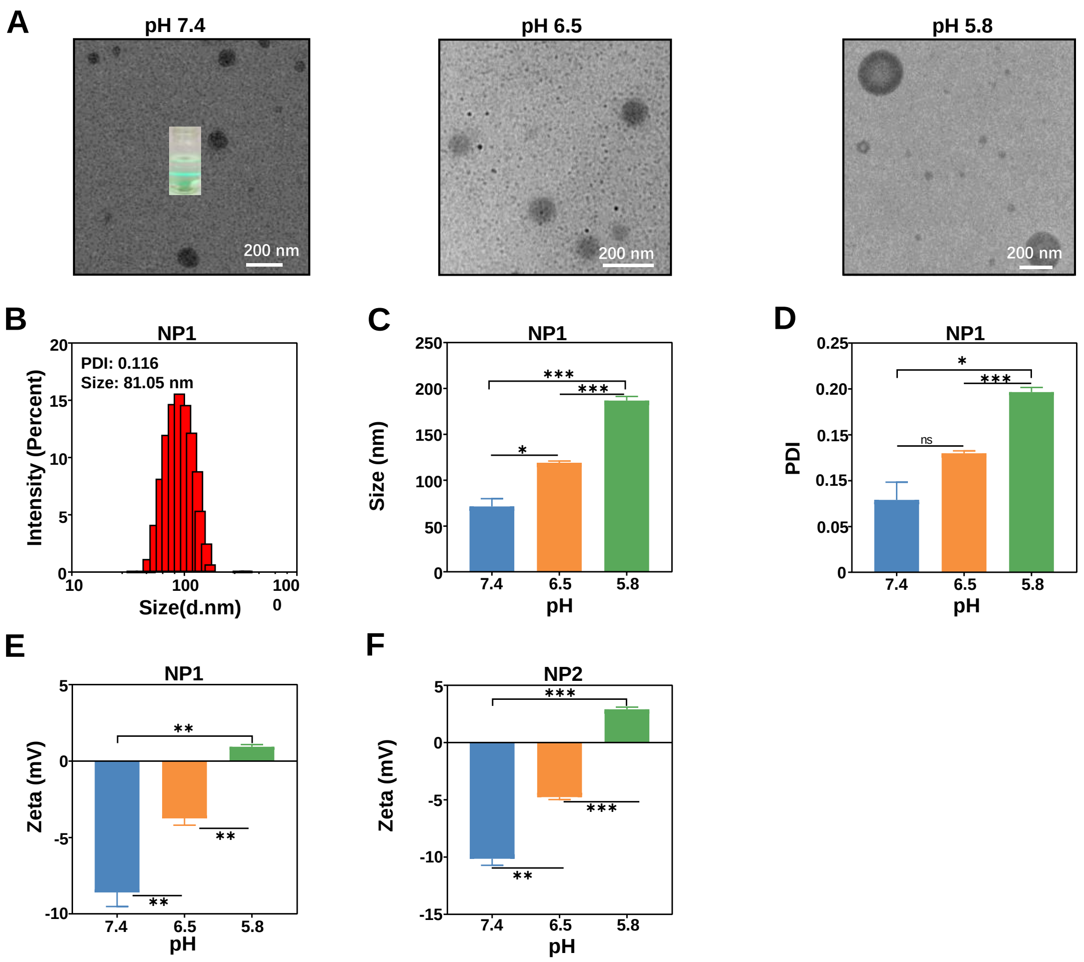

Figure S4. Characterization of **NP1**. (A) Transmission electron microscopy image of **NP1** upon incubation at various pH levels. (B) Dynamic light scattering distribution of **NP1**. (C) Change in hydrodynamic diameter of **NP1** at various pH levels. (D) Change in polydispersity of **NP1** at various pH levels. (E) Change in the zeta potential of **NP1** at various pH levels. (F) Change in the zeta potential of **NP2** at various pH levels. Each experiment was repeated three times. C-F) Data represent mean ± standard deviation (SD) from n independent experiments (n=3). Data is presented as mean ± standard deviation. Statistical significance was determined using an analysis of variance (ordinary one-way ANOVA) test. The p-values were found to the following range: ns = no statistical difference, * $p < 0.05$, ** $p < 0.01$, *** $p < 0.001$.

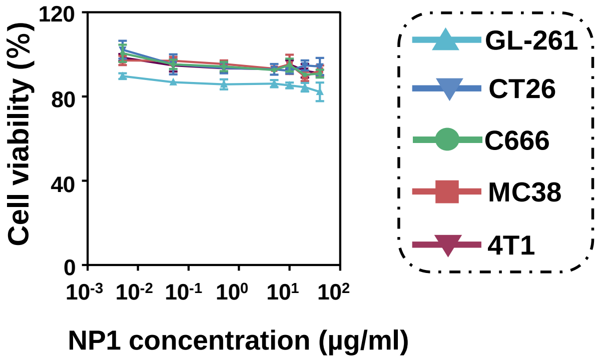

Figure S5. Drug-response curves of **NP1** against colorectal (CT26 and MC38), breast (4T1), nasopharyngeal carcinoma (C666), and glioma (GL261) cancer cells.

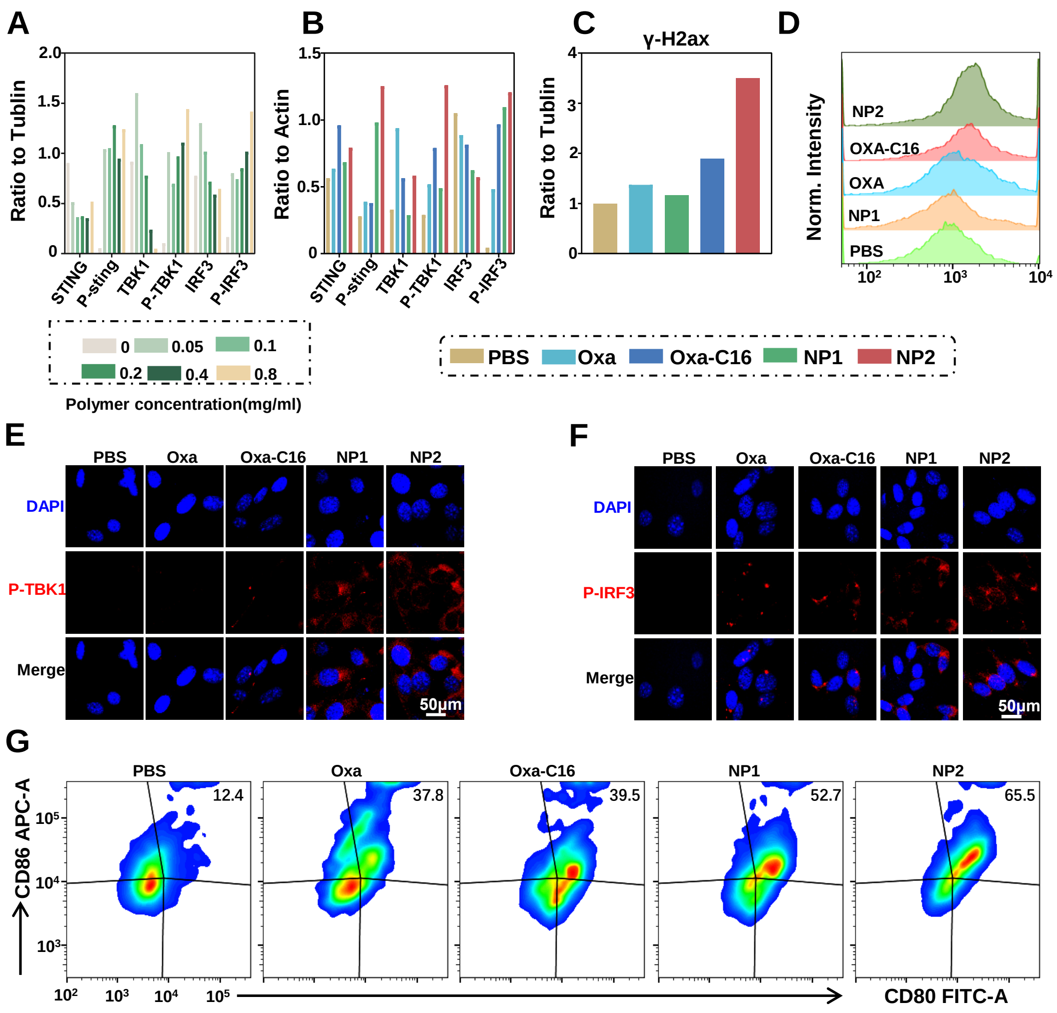

Figure S6. Activation of the STING pathway upon various treatments. (A) Comparison of the expression levels of STING pathway associated proteins in CT26 cells upon concentration dependent treatment with **NP1**. Data was determined by Western Blot analysis from Figure 4B. (B) Comparison of the expression levels of STING pathway associated proteins in CT26 cells upon various treatments. Data was determined by Western Blot analysis from Figure 4C. (C) Comparison of the expression level of γ-H2A in CT26 cells upon various treatments. Data was determined by Western Blot analysis from Figure 4D. (D) Comparison of the expression of γ-H2A in CT26 cells upon treatment with Oxa, Oxa-C16, **NP1**, or **NP2** determined by flow cytometry. (E) Immunofluorescence confocal laser scanning microscopy images of P-TBK1 upon various treatments. (F) Immunofluorescence confocal laser scanning microscopy images of P-IRF3 upon various treatments. **(**G) Maturation of mouse bone marrow-derived dendritic cells determined by flow cytometry. Analysis of CD11c+ lymphocytes gated with CD80+ CD86+ dendritic cells.

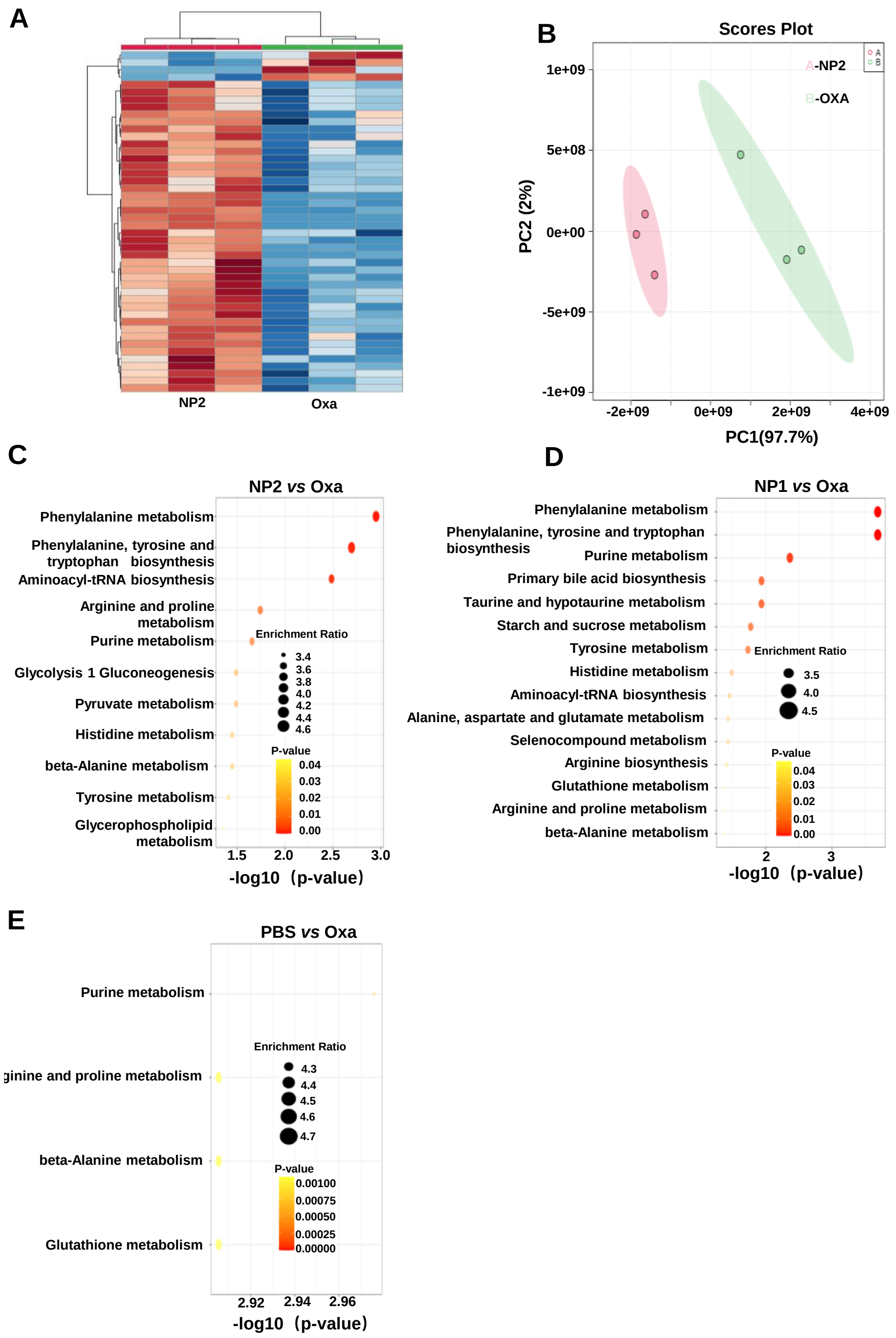

Figure S7. Metabolomics analysis of CT26 cells treated with Oxa, **NP1**, and **NP2**. (A) Heat map of the levels of identified metabolites upon treatment with Oxa and **NP2**. (B) Metabolite distribution upon treatment with Oxa and **NP2**. (C-D) KEGG enrichment analysis of the treatment with Oxa, **NP1**, and **NP2**. The size of the points corresponds to the enrichment ratio and the color of the point corresponds to the relevant p-value.

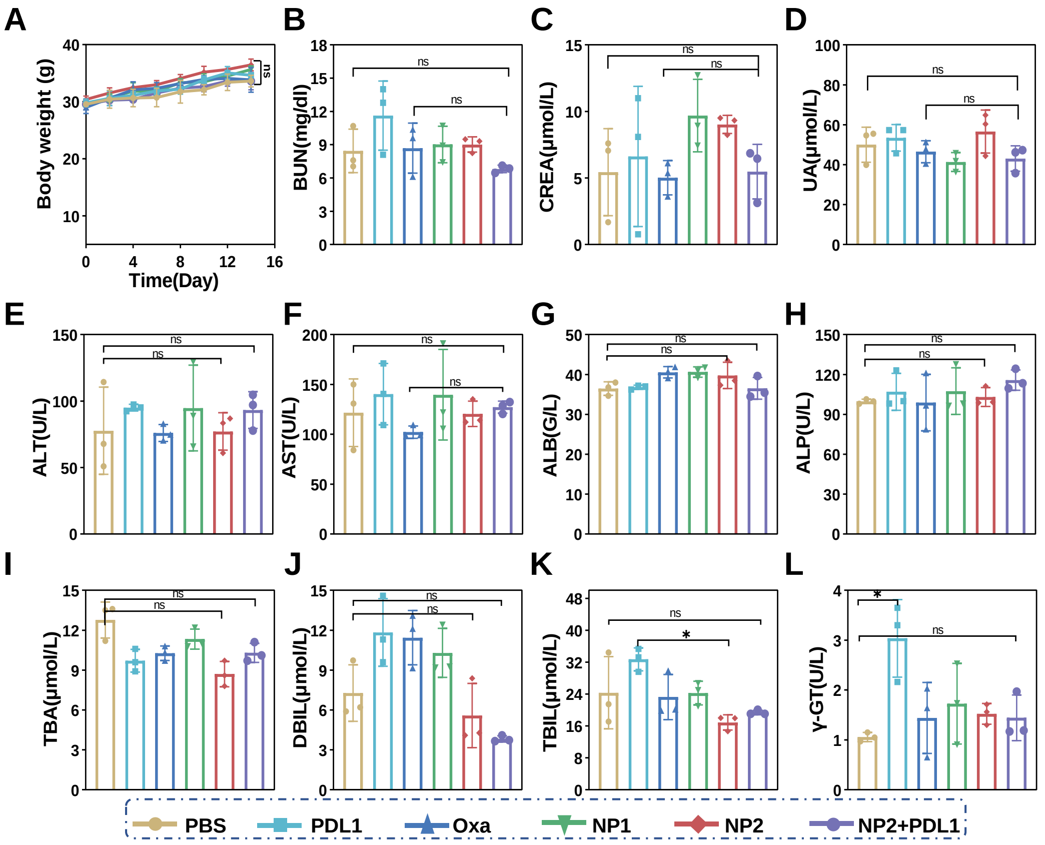

Figure S8. Biocompatibility study of healthy Kunming mice upon various treatments. (A) Changes in body weight upon various treatments in a period of 15 days. (B-L) Levels of the liver and kidney function indexes. Each experiment was repeated three times. (B-L) Data represent mean ± standard deviation (SD) from n independent experiments (n=3). Statistical significance was determined using an analysis of variance (ordinary one-way ANOVA) test. The p-values were found to the following range: ns = no statistical difference. * $p < 0.05$.

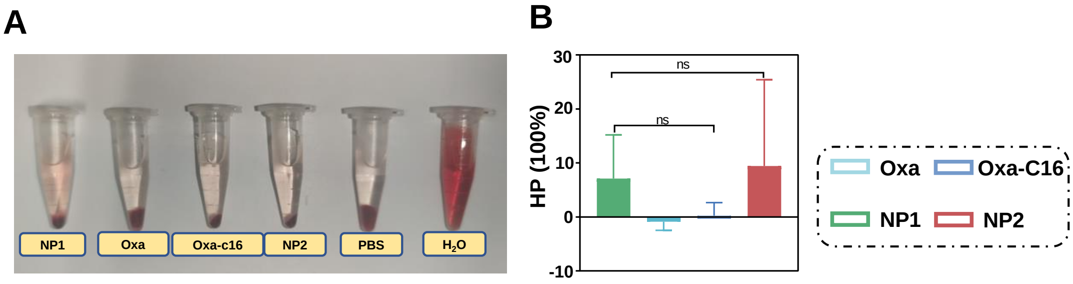

Figure S9. Hemolysis study. (A) Photograph of the evaluation of the hemolysis in the serum of the mice upon treatment. (B) Percentage of hemolysis in the serum of mice upon various treatments. Each experiment was repeated three times. Data represent mean ± standard deviation (SD) from n independent experiments (n=3). Statistical significance was determined using an analysis of variance (ordinary one-way ANOVA) test. The p-values were found to the following range: ns = no statistical difference.

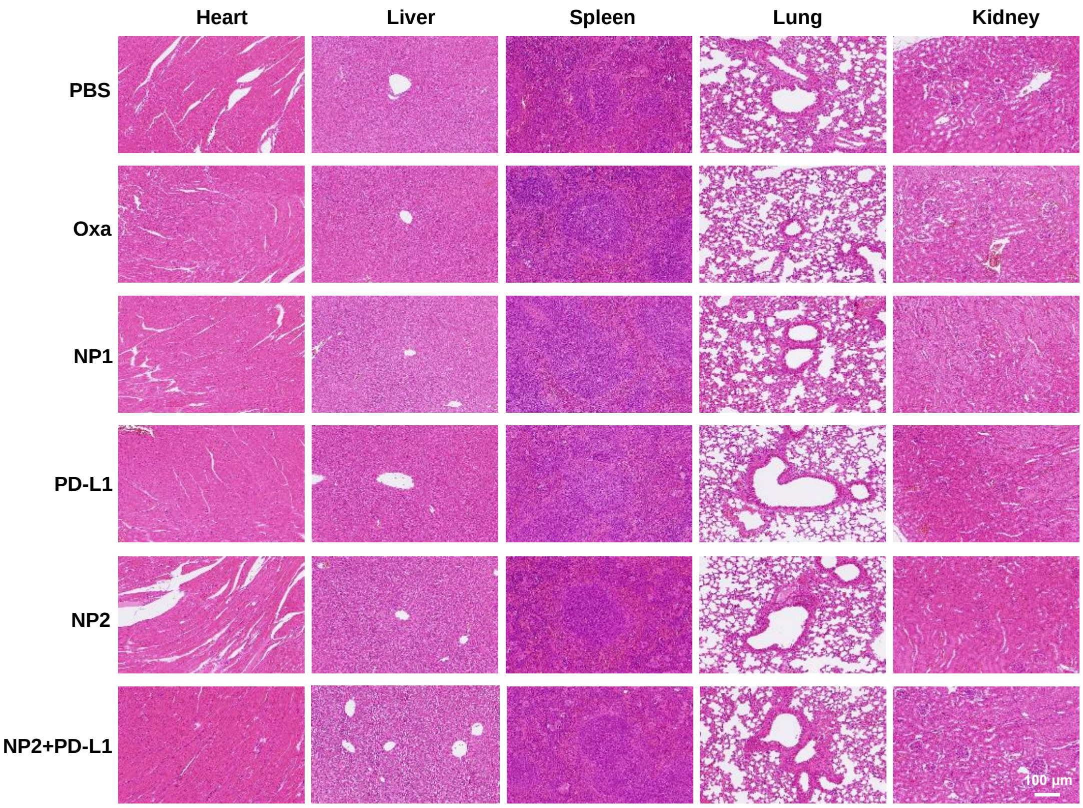

Figure S10. H&E study of major organs for systematic toxicity study.

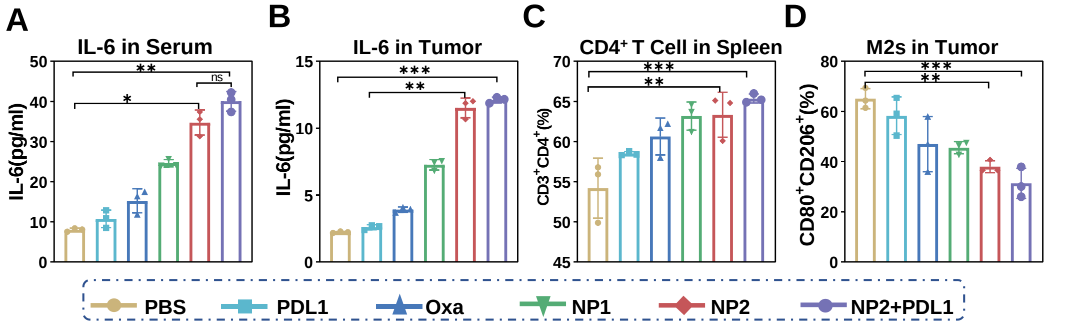

Figure S11. Activation of the STING pathway upon various treatments on a CT26 xenograft mouse model. (A) Levels of IL-6 in the serum upon treatment. (B) Levels of IL-6 in the tumor upon treatment. (C) Levels of $CD4^+$ T cells in the spleen upon treatment. (D) Percentage of M2 type macrophages upon treatment. Each experiment was repeated three times. A-D) Data represent mean ± standard deviation (SD) from n independent experiments (n=3). Statistical significance was determined using an analysis of variance (ordinary one-way ANOVA) test. The p-values were found to the following range: ns = no statistical difference, * $p < 0.05$, **$p < 0.01$, ***$p < 0.001$.

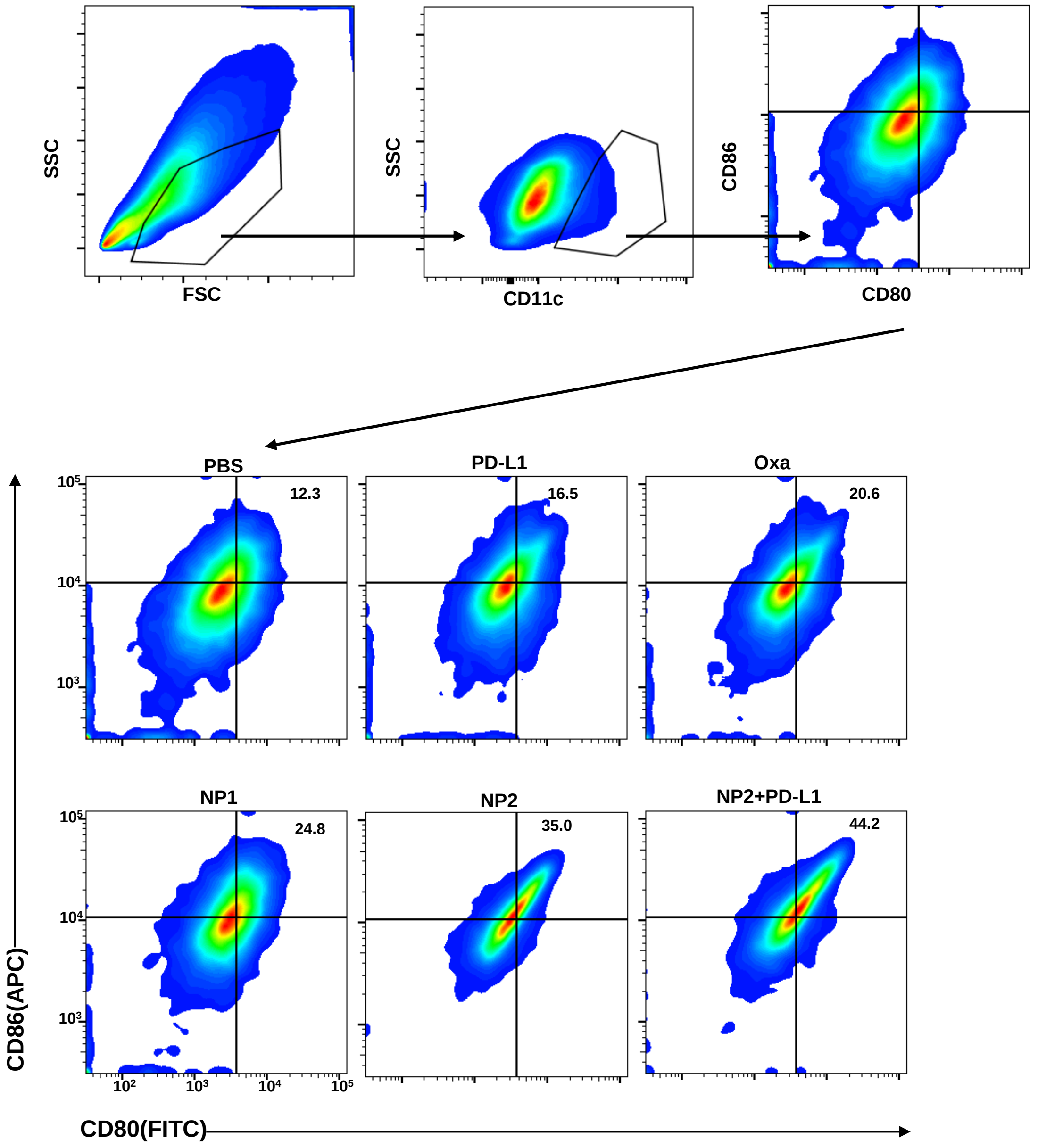

Figure S12. Flow cytometric analysis of CD80+ CD86+ DC gated CD11c+ cells in tumors.

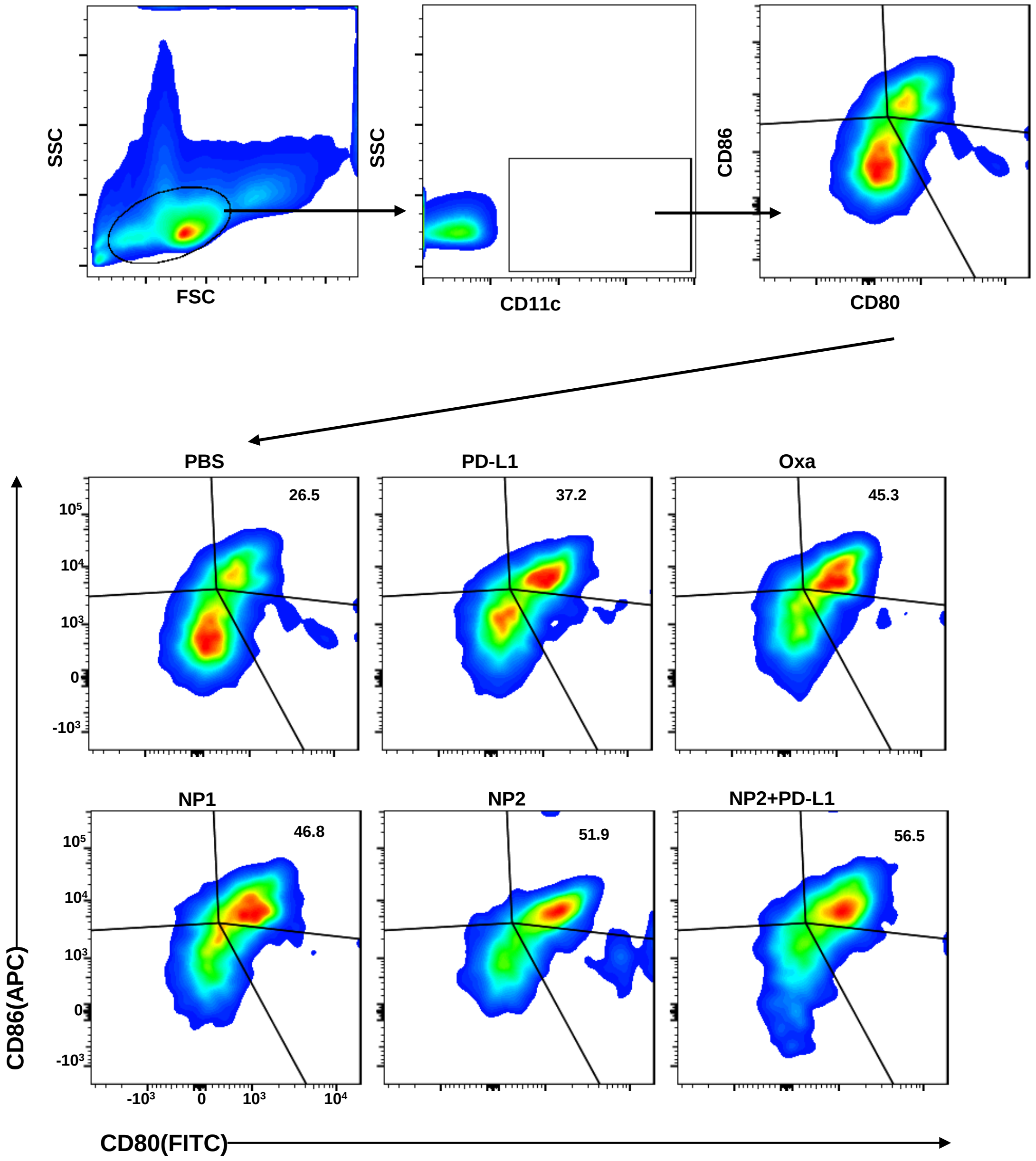

Figure S13. Flow cytometry analysis strategy of CD80+ CD86+ DC gating CD11c+ cells in lymphocytes.

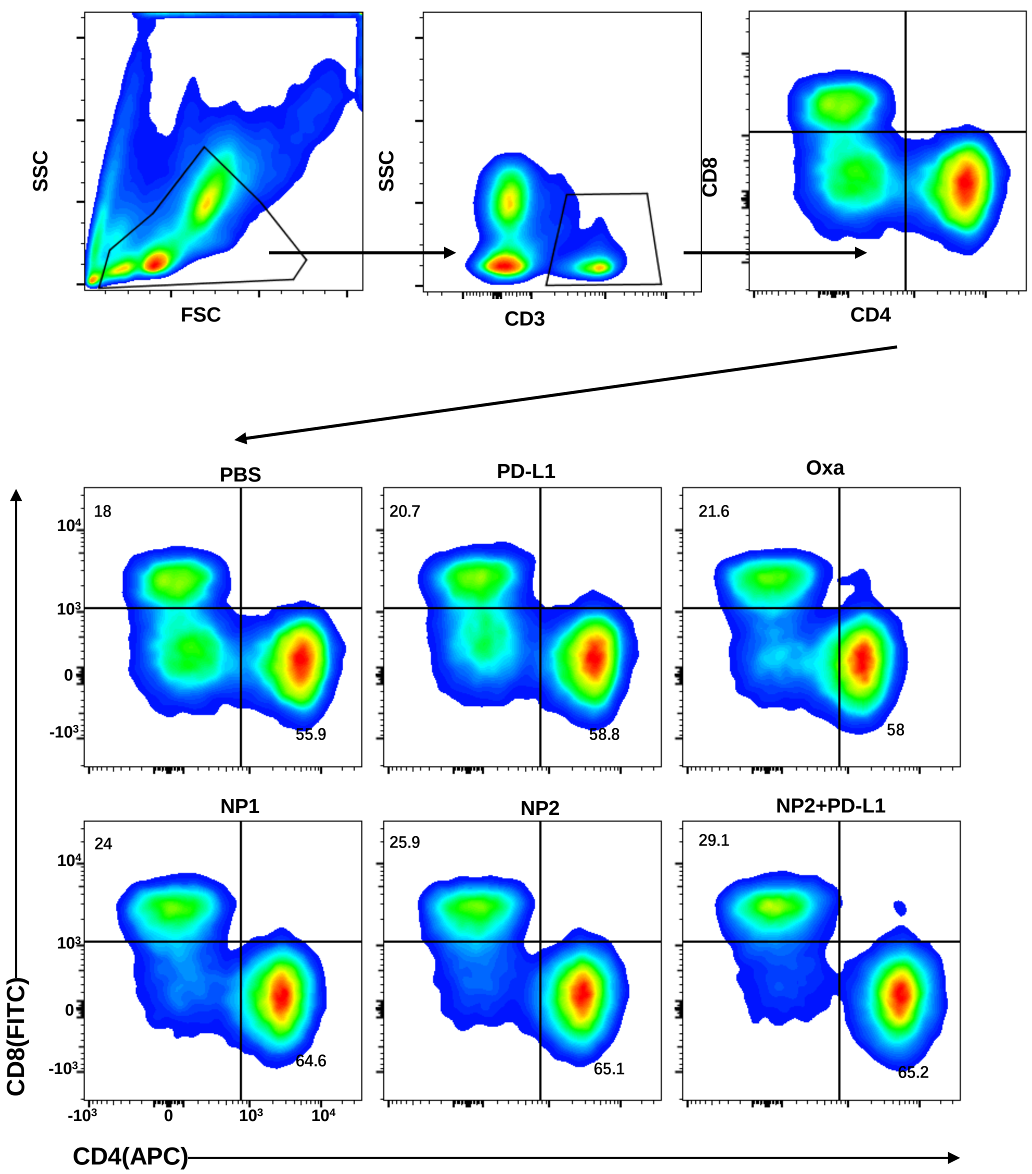

Figure S14. Flow cytometric analysis of gated CD3c+ cells by CD8+ and CD4+T cells in spleen.

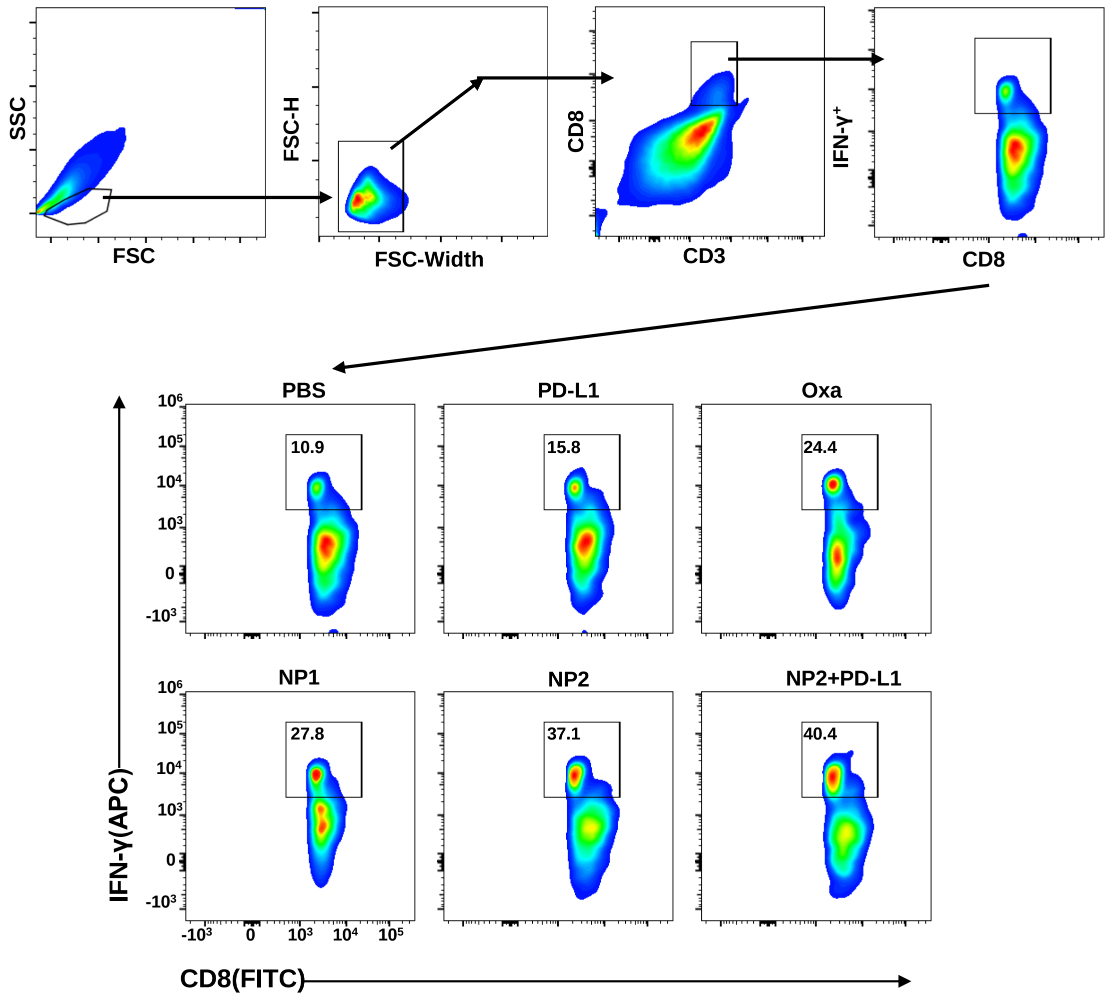

Figure S15. Flow cytometric analysis of CD3c+ cells gated by CD8+ IFN-γ+ T cells in tumors.

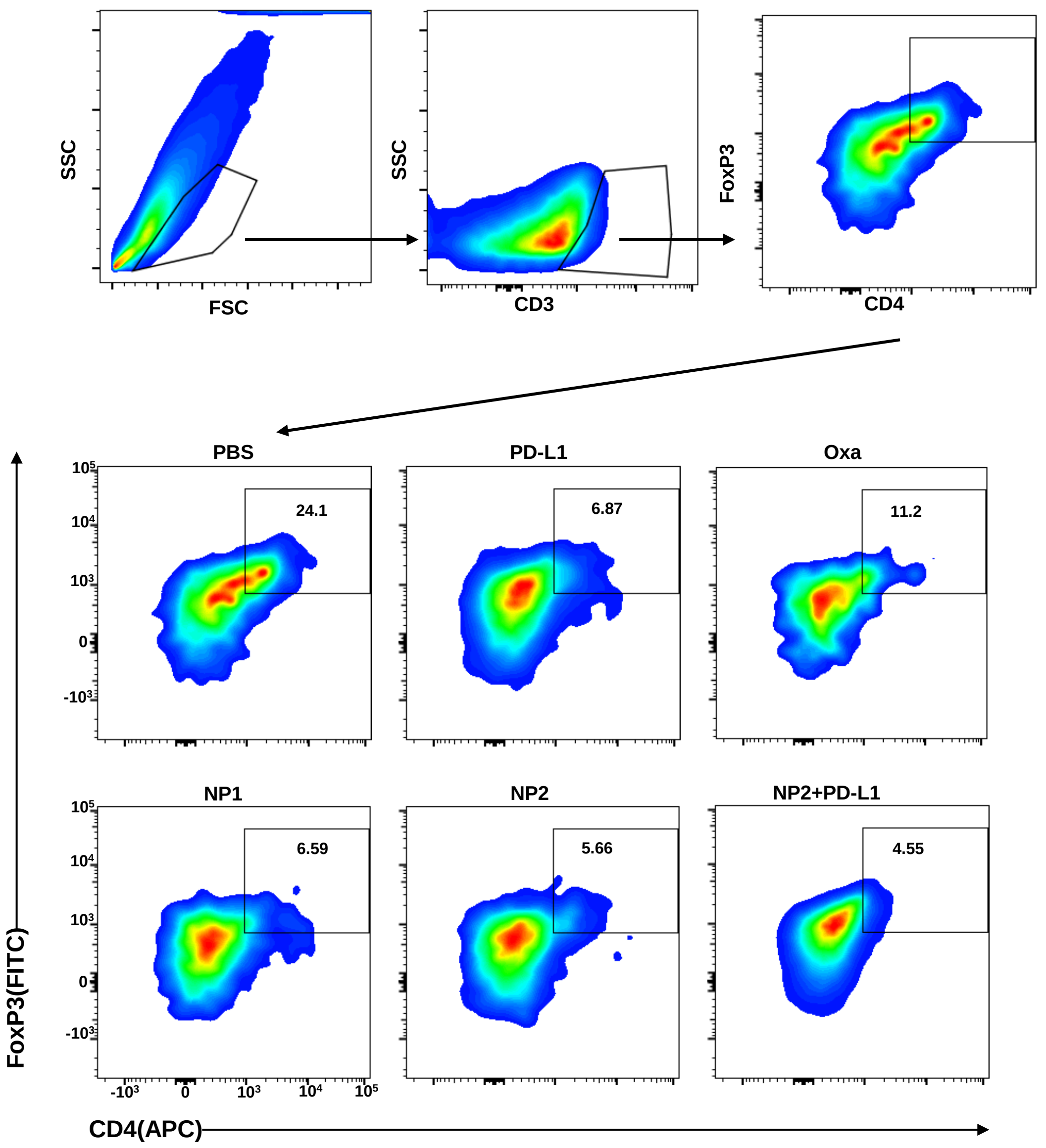

Figure S16. CD3c+ cell gated flow cytometric analysis of Tregs cells in tumors.

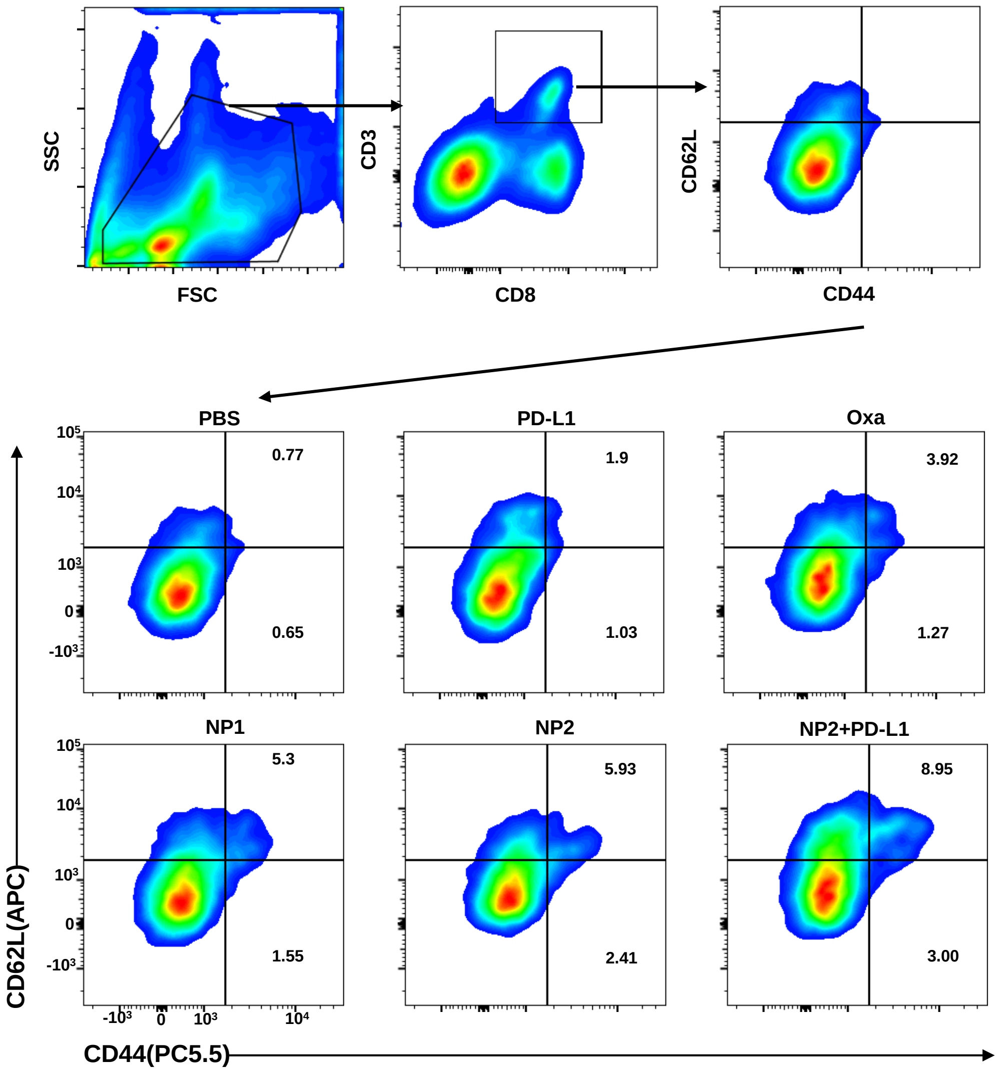

Figure S17. Flow cytometric analysis of CD3c+ cells gated by TCM and TEM in tumors.

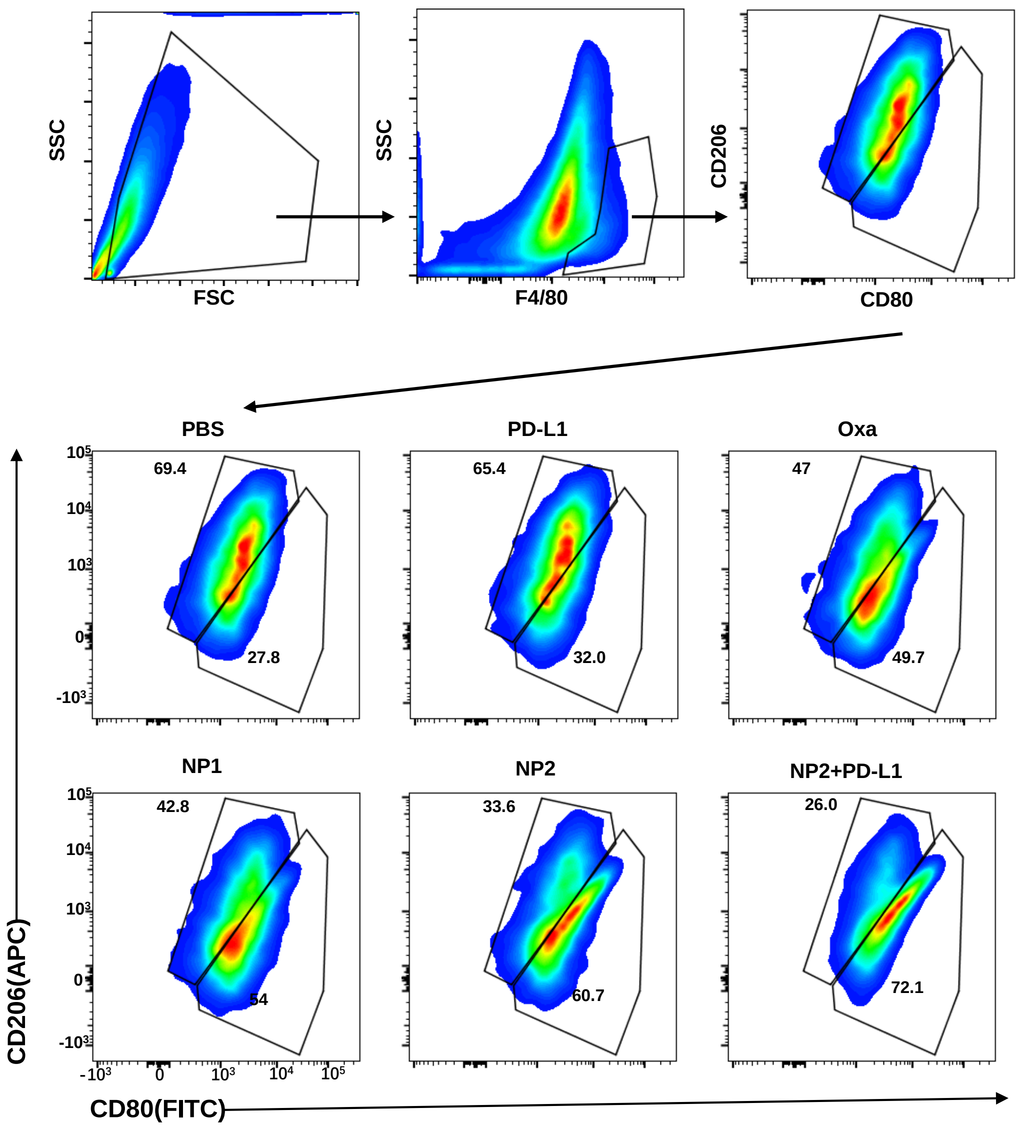

Figure S18. Gated strategy for flow cytometry analysis of TAMs cells.

Table S1. The IC_{50} values and standard deviations for the cytotoxicity (μM).

**Drug**	C666	4T1	GL-261	CT-26	MC38
Oxa	42.8±3.81	1.16±0.30	0.90±0.35	3.04±0.51	2.486±0.58
Oxa-C16	20.227±1.13	2.96±0.71	1.73±0.33	14.04±4.55	14.03±1.72
**NP2**	6.763±0.30	0.46±0.02	1.25±0.47	22.34±5.59	7.28±0.99

References

1. Feng, B.; Zhou, F.; Xu, Z.; Wang, T.; Wang, D.; Liu, J.; Fu, Y.; Yin, Q.; Zhang, Z.; Yu, H.; Li, Y., Versatile Prodrug Nanoparticles for Acid-Triggered Precise Imaging and Organelle-Specific Combination Cancer Therapy. *Advanced Functional Materials* **2016,** *26* (41), 7431-7442.
